# Novel PEGylated cholephytosomes for targeting fisetin to breast cancer: in vitro appraisal and in vivo antitumoral studies

**DOI:** 10.1007/s13346-023-01409-5

**Published:** 2023-08-30

**Authors:** Sara M. Talaat, Yosra S. R. Elnaggar, Mennatallah A. Gowayed, Samar O. El-Ganainy, Maram Allam, Ossama Y. Abdallah

**Affiliations:** 1 Department of Pharmaceutics, Faculty of Pharmacy, Alexandria University, Alexandria, Egypt; 2 Head of International Publication and Nanotechnology Center INCC, Department of Pharmaceutics, Faculty of Pharmacy and Drug Manufacturing, Pharos University of Alexandria, Alexandria, Egypt; 3 Department of Pharmacology and Therapeutics, Faculty of Pharmacy, Pharos University in Alexandria, Alexandria, Egypt; 4 Department of Pathology, Faculty of Medicine, Alexandria University, Alexandria, Egypt

**Keywords:** Fisetin, Cholephytosomes, Stearylamine, Hyaluronic, Breast cancer

## Abstract

Fisetin (FIS) is a multifunctional bioactive flavanol that has been recently exploited as anticancer drug against various cancers including breast cancer. However, its poor aqueous solubility has constrained its clinical application. In the current work, fisetin is complexed for the first time with soy phosphatidylcholine in the presence of cholesterol to form a novel biocompatible phytosomal system entitled “cholephytosomes.” To improve fisetin antitumor activity against breast cancer, stearylamine bearing cationic cholephytosomes (mPHY) were prepared and furtherly modified with hyaluronic acid (HPHY) to allow their orientation to cancer cells through their surface exposed phosphatidylserine and CD-44 receptors, respectively. In vitro characterization studies revealed promising physicochemical properties of both modified vesicles (mPHY and HPHY) including excellent FIS complexation efficiency (˷100%), improved octanol/water solubility along with a sustained drug release over 24 h. In vitro cell line studies against MDA-MB-231 cell line showed about 10- and 3.5-fold inhibition in IC50 of modified vesicles compared with free drug and conventional drug-phospholipid complex, respectively. Preclinical studies revealed that both modified cholephytosomes (mPHY and HPHY) had comparable cytotoxicity that is significantly surpassing free drug cytotoxicity. TGF-β1and its non-canonical related signaling pathway; ERK1/2, NF-κB, and MMP-9 were involved in halting tumorigenesis. Thus, tailoring novel phytosomal nanosystems for FIS could open opportunity for its clinical utility against cancer.

**Graphical Abstract:**

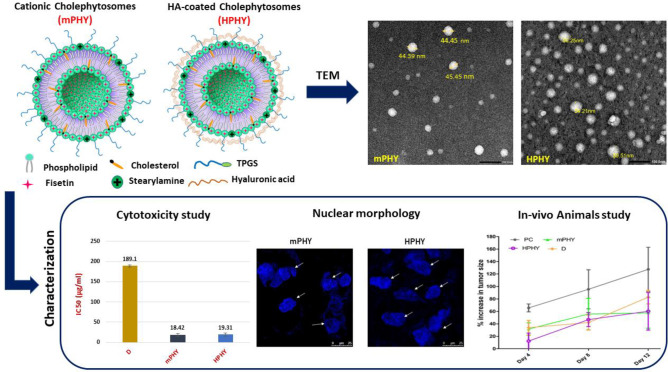

**Supplementary Information:**

The online version contains supplementary material available at 10.1007/s13346-023-01409-5.

## Introduction

In spite of the growing global health-care interest and also population awareness of breast cancer (BC), it is ranked in the recent years as the most common cancer diagnosed in women and the fifth leading cause of cancer death [1]. Because of BC heterogeneity, its management always require combination of different therapeutic approaches including chemotherapy [2]. In fact, the commercially available chemotherapeutic agents are suffering of inadequate tumor selectivity and specificity, rapid clearance, and resistance of cancer cells in addition to serious systemic side effects [3]. Such limitations resulted in poor patient compliance and week systemic response. Therefore, looking for other effective therapeutic alternatives with higher safety profile is considered one of the major scientific concerns nowadays.

Recently, a bioactive flavonoid known as fisetin (FIS) has gained the attention of scientific community due to its high potency along with its low toxicity. It is one of the major polyphenolic flavonoids available in concentration range of 2–160 µg/g in various fruits and vegetables such as strawberry, apple, grape, persimmon, onion, and cucumber [4]. It has multifunctional activities against different pathological conditions including cancer [5]. Against breast cancer, FIS exhibited cytotoxicity against most breast cancer subtypes including triple negative breast cancer (TNBC) [6] in addition to estrogen receptor and human epidermal growth factor receptor 2-overexpressing breast cancer cell [7]. It could inhibit tumor growth through induction of apoptosis, cell cycle arrest in addition to interference with angiogenesis, invasion, and also metastasis through multiple molecular and signaling pathways [8–10].

In spite of the plentiful biological activities of FIS as anticancer phytomedicine, its clinical applications are still limited due to its poor aqueous solubility (less than 1 mg/ml) constraining its oral bioavailability and also its parenteral administration [11]. Therefore, various strategies have been adopted to surmount FIS pharmaceutical handicaps including liposomes [12], cochleates [13], polymeric micelles [14], nanoemulsion [15], co-crystals [16], and cyclodextrins complexation [17]. However, most of these approaches have been utilized to surpass mainly the problems associated with solubility, oral bioavailability, and in vitro activity of FIS. Meanwhile, lacking the preclinical pharmacological studies to confirm its systemic therapeutic efficacy. On the other hand, FIS-encapsulated delivery systems that have been in vivo appraised for their therapeutic activity are suffered from numerous challenges restricting their clinical applications. For example, liposomes exhibited unsatisfied FIS encapsulation efficiency [12]. Polymeric-based nanoparticles [14] and surfactant-based micellar nanosystems [18] are complex in their preparation involving multiple step processing beside to using sometimes toxic solvents and/or surfactants. Additionally, there are limited data available concerning their biodegradability, biocompatibility, and immunogenicity [19]. That is why, none of FIS pharmaceutical nanoformulations has passed the research phase and stepped forward to reach the commercial phase. Looking forward to novel delivery systems for fisetin that could solve the major drawbacks facing the developed nanocarriers is considered a priority for the scientific research.

Of late, complexation of herbal drugs with phospholipids such as soy phosphatidylcholines has gained great global research interest. The formed complex can be either encapsulated in various nanocarriers with higher loading efficiency [20, 21] or even self-assembled in aqueous vehicle to form liposomal like nanovesicles known as phytosomes or herbosomes. Because herbal drug itself is chemically bound to phospholipid, phytosomes are reported to surpass other lipid-based nanocarriers such as liposomes where they showed superior physicochemical properties including better physical stability, higher entrapment efficiency, lower susceptibility for drug leakage, or its expulsion form the delivery system [22]. Furthermore, the amphiphilic phospholipid which is the main forming unit of phytosomes could improve aqueous solubility of the complexed phytochemicals and enhance their permeability through biological membranes emphasizing their biological activities. The role of phospholipids against cancer should be also highlighted where they are known as natural antioxidants in addition to their potential to inhibit tumor growth and metastasis [23].

Although “PHYTOSOME®” technology is considered as breakthrough for the clinical validity of various herbal polyphenols [24], such innovative approach has not been so far employed for fisetin. Thus, “cholephytosomes” were formulated, for the first time, through integration of cholesterol with fisetin-phospholipid complex in attempt to develop novel phytosomal system with modified physicochemical properties. Inspired by liposomes, cholesterol could improve the in vitro and in vivo stability of vesicles through decreasing their vesicular permeability and improving their resistance to aggregation [25]. Furthermore, cholesterol could dramatically affect cellular uptake of lipid vesicles [26, 27] through promoting their rigidity [28]. In case of cancer, cholesterol has been recently highlighted to play a pivotal role through enhancing internalization of cytotoxic drugs via specific receptor mediated uptake mechanism in various types of cancer [29, 30].

In order to emphasize in vivo efficacy against breast cancer along with minimizing their systemic toxicity, active targeting strategy should be adopted through functionalization of cholephytosomes surface with specific targeting ligands. Actually, the modified nanovesicles should be selectively oriented to receptors or markers that are abundantly expressed on surface of breast cancer cells more than normal cells. In the current study, targeting surface CD-44 receptors and also externalized phosphatidylserine would be exploited as promising targeting approaches in attempt to potentiate FIS anticancer activity against breast cancer. Targeting CD-44 receptors is a well-known approach that has been employed against different cancers including breast cancer [31]. Therefore, hyaluronic acid (HA) was utilized as a CD-44 targeting ligand to decorate cholephytosomes surface for fisetin breast cancer targeted delivery [32].

Most recently, charge-dependent targeting approach has been spotlighted for targeting tumor cells via their surface exposed phosphatidylserine [33]. Phosphatidylserine (PSe) is the most abundant anionic phospholipid confined to the inner membrane leaflet as part of the normal asymmetrical distribution of phospholipids. Yet, it can be externalized in most viable and non-apoptotic tumor cells acting as immunosuppressive facilitating tumor growth and metastasis [34]. In light of this, Novel PSe-selective therapies have been investigated in preclinical and clinical studies against different types of tumors including breast cancer such as proteoliposomal nanovesicles of saposin-C coupled with dioleoylphosphatidylserine (SapC-DOPS) [35]. In the current work, stearylamine bearing cationic cholephytosomes were developed to effectively target breast tumor cells and also to improve cellular uptake of FIS through selective interaction with anionic phosphatidylserine exposing on the surface of cancer cells.

In this context, the current study aims to elaborate for the first time a novel biocompatible self-assembled phospholipid complex integrated with cholesterol to produce fisetin cholephytosomes that can circumvent FIS drawbacks. Then, stearylamine bearing cationic cholephytosomes were developed and surface modification with hyaluronic acid was furtherly carried out in aim to improve FIS delivery to breast cancer cells. To the best of our knowledge, this study is considered the first to elucidate and compare the impact of using cationic stearylamine bearing phytosomes and CD-44 targeting phytosomes against breast cancer. Different assessment techniques were conducted for the designed vesicles to assess their in vitro physicochemical characterizations. In addition, appraisal of antitumor activity of the developed cationic FIS-cholephytosomes versus CD-44 targeting cholephytosomes was established using in vitro breast cancer cell lines and in vivo animal model.

## Materials and methods

### Materials

Fisetin (purity 98%) was purchased from X’ian Le Sen Bio-Technology Co., Ltd (China). Soy phosphatidylcholine (Lipoid S100) was purchased from Lipoid (Ludwigshafen, Germany). D-α-Tocopheryl polyethylene glycol 1000 succinate (TPGS) was purchased from Baoji Guokang Bio-Technology Co., Ltd (China). Hyaluronic acid (Mwt 10,000–40,000 Da), purity (96%) was a kind gift from Orchidia pharmaceuticals (Egypt). Cholesterol was a kind gift from The Nile for Pharmaceuticals & Chemical Industries (Cairo, Egypt). DAPI stain, annexin-v-FITC/propidium iodide kit, fetal bovine serum (FBS), and Dulbecco’s modified eagle medium (DMEM); composed of inorganic salts, amino acids vitamins, and others were purchased from Sigma-Aldrich (St. Louis, Missouri, USA). Penicillin and streptomycin solution (100 U/ml each) (BioWhittaker®, Lonza, Belgium). 3-[4, 5-dimethylthiazol-2-yl]-2, 5-diphenyl tetrazolium bromide (MTT) was purchased from Serva (Heidelberg, Germany). Human breast cancer cell line (MDA-MB-231) was obtained from the American Type Culture Collection (ATCC, MD, USA). Ehrlich ascites carcinoma (EAC) parent line was supplied from National Institute of Cancer, Cairo University, Egypt. All other chemicals and reagents used were of analytical grade.

### Preparation of fisetin–cholephytosomes

FIS-cholephytosomes were prepared using ethanol injection method. Initially, equimolar concentrations of fisetin and soy phosphatidylcholine Lipoid S100 were dissolved in minimum amount of ethanol. Then, the reaction mixture was kept under continuous mild magnetic stirring at 400 rpm for 1 h at 40–45 °C to allow complexation between the drug and phospholipid. Afterwards, cholesterol (Chol) was added to the reaction mixture under stirring until dissolved completely in the mixture. The reaction mixture was consequently injected to 8-ml deionized water at 40–45 °C under same magnetic stirring rate to allow self-assembly into cholephytosomes. Finally, the residual organic solvents were removed using rotary evaporator under vacuum at 40 °C [36]. For proper size reduction, the developed cholephytosomes were exposed to ultrasonication in ice-bath for 5 min (10 s on and 10 s off) at 60% amplitude using a probe sonicator.

### Preparation of active targeting PEGylated fisetin cholephytosomes

Phosphatidylserine targeting strategy was employed through preparation of cationic PEGylated cholephytosomes using positively charged lipid (stearylamine; SA) and PEGylated surfactant (tocopheryl polyethylene glycol 1000 succinate; TPGS). Also, decoration of the surface of prepared cationic cholephytosomes with hyaluronic acid (HA) was furtherly carried out to prepare the CD-44 targeting cholephytosomes (HPHY).

To prepare cationic cholephytosomes (mPHY), predetermined weights of stearylamine and TPGS were added together with cholesterol (Chol) to the reaction mixture of fisetin (FIS) and soy phosphatidylcholine (SPC) for 1 h before injection into aqueous vehicle under stirring (400 rpm) at 40–45 °C. Afterwards, self-assembly of the developed complex into cholephytosomes followed by organic solvent evaporation and proper size reduction was conducted as previously described for preparation of cholephytosomes. Eventually, surface decoration of cationic PEGylated cholephytosomes with selected anionic biopolymer (HA) was accomplished through the titration of 1 ml of positively charged modified cholephytosomes with different volumes of hyaluronic acid solution (5 mg/ml) under mild stirring (400 rpm) for 2 h. The minimum volume of HA solution capable of inverting the surface charge to positive with accepted zeta potential was selected as the optimum volume.

### Characterization of PEGylated fisetin cholephytosomes

#### Complexation efficiency (% CE)

Complexation efficiency was assessed based on the solubility difference of pure FIS and FIS-SPC complex in chloroform. Thin film of the complexed FIS equivalent to 25 mg FIS was dissolved in 3 ml chloroform with vigorous vortex to disperse it properly. The mixture was then centrifuged at 6000 rpm for 5 min to precipitate any free non-complexed fisetin. Subsequently, the quantity of FIS in the complex was determined in the supernatant and any precipitate was discarded. To measure CE%, proper dilution with ethanol was carried out followed by spectrophotometric assay of FIS content at *λ* 360 nm. The following equation was used to calculate complexation efficiency (% CE):

% CE = $$\frac{\mathrm{W \;}(\mathrm{Complexed \;FIS})}{\mathrm{W\; }(\mathrm{Total \;FIS \;added})}*100$$  

#### Octanol–water solubility study

The solubility of Chol/SA/TPGS-modified FIS-SPC complex was determined and compared with solubility of pure fisetin using shake flask method. In brief, excess FIS or modified FIS-SPC complex was added to 3 ml of deionized water or n-octanol in sealed glass containers. Then, samples were shaken for 24 h at 25 °C and centrifuged for 5 min at 4000 rpm to remove excessive insoluble drug. Then, the supernatant was filtered through a 0.22-μm membrane filter followed by proper dilution with ethanol. Fisetin solubility from each sample was determined in each vehicle (water and n-octanol) spectrophotometrically at *λ* 360 nm in triplicates[37].

#### Particle size, ζ-potential, and polydispersity index

Dynamic light scattering technique (DLS) was applied to measure particle size (PS), polydispersity index (PDI), and ζ-potential (ZP) of modified cholephytosomes (mPHY and HPHY). The samples were measured in triplicates, and the results were expressed as mean size ± SD.

#### Fourier transform infrared spectroscopy (FTIR)

The complex formation between fisetin and soy phosphatidylcholine (Lipoid® S100) was investigated through IR spectra spectroscopy technique using FTIR spectrometer (PerkinElmer Inc). Samples were mixed with dry crystalline KBr in a ratio of 1:100, and pellets were prepared. A spectrum was collected for each sample within the wave number region 4000–400 cm^−1^. Samples under investigation were FIS, SPC, and lyophilized formulations of modified fisetin cholephytosomes.

#### Transmission electron microscopy (TEM)

The morphology of modified fisetin cholephytosomes (mPHY and HPHY) was investigated using transmission electron microscopy (TEM; JEM-100 CX electron microscope, JEOL, Japan). Aqueous dispersion of freshly prepared samples was diluted with filtered deionized water (1:10) and sonicated for 1 min. Then a drop of the dispersion was placed on a copper grid, and excess suspension was removed by filter paper. The samples were subsequently stained with saturated solution of uranyl acetate followed by air-drying before imaging under high voltage of 80 kV.

#### In vitro release study

The standard dialysis technique was applied to investigate in vitro release profiles of different FIS–cholephytosomal dispersions. FIS aqueous suspension prepared in 0.5% sodium carboxymethylcellulose solution and its drug solution (50% w/w mixture of PEG 400 and water) were included for comparative studying against FIS nanovesicles [20]. All the tested samples were placed into a sealed dialysis bag (MWCO 12,000–14,000) in a final concentration equivalent to 1 mg/ml of fisetin. After sealing the dialysis bags well, they were immersed into 50 ml of release medium (phosphate-buffered saline; PBS, pH 7.4) containing 20% alcohol to assure sink condition. The experiment was carried out at 37 °C ± 0.5 °C and 100 rpm using a thermostatic shaking water bath. Aliquots of 1 ml were withdrawn from release medium at predetermined time intervals (0.5, 2, 4, 6, 8, and 24 h), then compensated with equal volume of fresh release medium. The withdrawn samples were subsequently filtered through 0.45-µm membrane filter, then measured spectrophotometrically against release medium at *λ* 320 nm [38]. Results—in triplicates—were represented as percentage cumulative release ± SD, and graph of % cumulative drug release versus time was plotted.

#### Hemocompatibility test

Hemocompatibility of modified cationic cholephytosomes (mPHY) and hyaluronic decorated cholephytosomes (HPHY) was studied as previously reported by Khatik et al. [39] with slight modifications to test their in vitro hemolytic activity against RBCs. Briefly, fresh blood collected from a healthy human volunteer was collected using heparinized vials and centrifuged at 2000 rpm for 15 min. Supernatant was discarded, and sediment RBCs were collected and washed twice with normal saline (0.9% w/v). The collected RBCs were then diluted with normal saline to obtain RBCs suspension (2% v/v). The optimized cholephytosomal nanoformulations were incubated with RBCs suspension in 1:1 volume ratio at 37 °C for 1 h with mild shaking (100 rpm). Afterwards, samples were centrifuged for 5 min at 3000 rpm, and supernatants were collected for hemoglobin content quantification by spectrophotometric analysis at *λ*_max_ 540 nm. Positive control (100% hemolysis) and negative control (0% hemolysis) were obtained by mixing RBCs suspension in 1:1 v/v with 1% Triton-X 100 or normal saline, respectively. % Hemolysis was calculated using the following equation:

$$\mathrm{\% \;Hemolysis }= \frac{\mathrm{A\; sample \;at\; }\lambda \;540-\mathrm{A\; negative\; control\; at\;} \lambda \;540}{\mathrm{A \;positive \;control \;at\;} \lambda \;540}*100$$

where, A sample is the absorbance of the sample; A negative control is the absorbance of the negative control (RBC in 0.9% normal saline); and A positive control is the absorbance of the positive control (RBC in 1% Triton X).

#### Short shelf-life stability of lyophilized FIS-cholephytosomes

Freshly prepared dispersions of the selected fisetin cholephytosomes were lyophilized using trehalose as cryoprotectant at concentration of 2% (w/v). The vials were frozen at − 20 °C for 48 h and were then placed in a lyophilizer. Lyophilization was performed at a pressure of 40 mbar and a shelf temperature of − 50 °C for 24 h. In order to test the stability of the lyophilized samples, the powder was reconstituted with 1-ml deionized water and subjected to bath sonication for 1 min. Then, samples were suitably diluted with deionized water where the reconstituted cholephytosomes were monitored for changes in particle size, PDI, zeta potential, EE%, and any change in the physical state initially and after 4 weeks of storage. The lyophilized particles were stored in a desiccator over CaCl_2_ at 25 ℃ until testing, and all measurements were done in triplicate.

### In vitroanticancer activity (cell line studies)

MDA-MB-231 cells were selected as a model of CD-44 receptors positive breast cancer cells, exposing PSe on their surface. Cells were allowed to grow in (DMEM)-high glucose in the presence of fetal bovine serum (FBS; 10% v/v) and antibiotics (100 U/ml penicillin and 100 μg/ml streptomycin). Their growth was maintained at 37 °C in an incubator with 5% CO_2_ atmosphere. All in vitro cell line studies were carried out in CERRMA (Center of Excellence for Research in Regenerative Medicine and its Applications), Faculty of Medicine, Alexandria University.

#### Cellular cytotoxicity assay

Cellular cytotoxicity against MDA-MB-231 was assessed using MTT assay. MDA-MB-231 cells were seeded at a density of (5 × 10^3^) in 96-well plate (Corning, NY) and allowed to adhere for 24 h. Then, they were treated with different concentrations of modified cholephytosomes (mPHY and HPHY). The impact of phospholipid complexation on FIS cytotoxicity was studied in comparison with free drug. Meanwhile, conventional phytosomes (PLX) were enrolled to study the effect of phytosomes modification on its overall efficacy. After incubation for 24 h, 100-μl fresh media containing 10-μl MTT solution (5 mg/ml) were added and incubated for another 3 h in CO_2_ incubator. Finally, 100 μl DMSO was added to dissolve the produced formazan crystals. Absorbance was measured at 570 nm using a microplate reader (BioTek Instruments, Inc, VT, USA). % Cell viability was determined according to the following equation [40]:

$$\%\;\mathrm V\mathrm i\mathrm a\mathrm b\mathrm i\mathrm l\mathrm i\mathrm t\mathrm y=\frac{\mathrm{A\;sample\;at\;}\lambda\;570}{\mathrm{A\;control\;at}\;\lambda\;570}\ast100$$

% Cell viability was calculated by comparing the optical density of each treated cells with control cells treated only by culture media. Furtheremore, IC50 was calculated using a GraphPad Prism 6, and the results were expressed as mean ± SD (*n* = 3).

#### Apoptosis assay (annexin-V-FITC/propidium iodide assay)

The apoptotic effect of fisetin and other fisetin phytosomal nanoformulations was investigated by annexin-V assay using flow cytomerty. Briefly, cells were incubated at a density of (2 × 10^5^) in 6-well plate (Corning, NY) and allowed to adhere in an incubator for 24 h at 37 °C. Next, cells were treated with 20 μg/ml FIS or equivalent dose of FIS cholephytosomal formulations for 24 h. Afterwards, cells were trypsinized, collected by centrifugation at 2000 rpm, and stained with annexin V-FITC and propidium iodide as per the manufacturer’s protocol. Analysis of apoptotic cells was done by 20,000 cells gating by flow cytometer (BD FACSCalibur™ flow cytometer (San Jose, USA). The experiment was done in triplicates (*n* = 3), and representative images were provided.

#### Nuclear morphology evaluation

Confocal microscopy was utlized to confirm the apoptotic effect of fisetin on MDA-MB-231 cells through visualization of the morphological changes on their nuclei. MDA-MB-231 cells were incubated at a density of (5 × 10^4^) in 6-well plate and cultured for 24 h to allow attachment under the condition of 5% CO_2_ at 37 °C [41]. The next day, cells were washed twice with PBS and then treated with either free fisetin or fisetin phytosomal dispersions (conventional phytosomes and modified cholephytosomes) at dose equivalent to 20 µg/ml in serum-free medium. After 24 h of uptake, the cells were washed three times with cold PBS, followed by fixation with 4% paraformaldehyde for 30 min. Cell nuclei were subsequently stained with 4′, 6-diamidino-2-phenylindole (DAPI) for another 20 min. Eventually, an LSM710 laser confocal microscope confocal microscopy (LEICA, DMi8, Mannheim/Wetzlar, Germany) (Zeiss, Germany) was used to observe and analyze the fluorescent signals in MDA-MB-231 cells. Images were taken using confocal laser scanning microscopy equipped with an argon laser and then processed using Leica Application Suite X (LAS X) Software.

### In vivoantitumor activity study

#### Animals

Female Albino Swiss CD1 mice (7–8 weeks of age, 25 ± 3 gm) were obtained from the animal house of Faculty of Pharmacy, Pharos University in Alexandria, Egypt, and kept under controlled temperature and humidity, allowed free access to food and water.

#### Induction of tumor

Derived from ascites fluid of Albino Swiss mice (8 to 10 days ascites tumor), Ehrlich ascites carcinoma cells were harvested. The freshly withdrawn fluid, approximately 10^6^ cells, was suspended in sterile saline and injected subcutaneously into the left mammary fat pad of Albino Swiss CD1 female mice. Tumor volume was assessed daily until reaching (100–150 mm^3^) [42]. Using digitalized vernier caliper, both axis of the tumor were measured, and tumor volume was calculated according to the following equation [43]:

V = (L × W^2^) × 0.5, where V tumor volume, L major axis, and W minor axis.

Tumor growth was monitored twice a week, and % tumor growth was calculated with respect to size of tumor at day zero treatment.

#### Experimental design

After injecting all mice with ascites carcinoma cells and reaching desired size, mice were randomly allocated into four groups (*n* = 7). The first group is untreated mice (PC-group); the second group is mice treated with fisetin drug solution (D-group); the third and fourth groups were mice treated FIS modified cholephytosomes (mPHY: cationic SA-bearing cholephytosomes and HPHY: hyaluronic decorated cholephytosomes). All FIS-formulations were administered in a dose of (10 mg/kg) intravenously in a biweekly fashion for 2 weeks. FIS drug solution was prepared in sterile normal saline (0.9%) containing (12.5%) propylene glycol, (12.5%) PEG 400, and (0.625%) Tween 80. Mice were sacrificed after the last dose of drug, and excised tumor was divided into two halves. First half was preserved in 10% formalin for histopathological examination, while the other half was stored at − 80 °C until further biochemical determinations.

#### Tumor volume determination

Tumor growth was monitored at three intervals; day 4, day 8, and day 12 during the treatment period. Percent increase was calculated with respect to day 0 treatment.

#### Enzyme-linked immunosorbent assay (ELISA)

Tumor tissues were analyzed for the protein levels of p-ERK1/2 (MyBioSource, USA) NF-κβ (FineTest, China) MMP9 (Novus Biologicals, USA) using ELISA as directed by manufacturer instructions. Values were normalized to total protein content in the sample determined by Beirut method.

#### Immunohistochemistry

Immunohistochemical staining was conducted to detect TGF-β1 (a marker of tumor proliferation) and E-cadherin (a marker of cell proliferation, invasion, and migration). Details of immunohistochemical method and quantification were described in supplementary[44, 45].

#### Histopathological examinations

Control and experimental mice were sacrificed and sections through breast mass, liver, and kidney were retrieved and washed with cold phosphate-buffered saline followed by fixation using 10% formalin. Paraffin-embedded blocks were prepared. After sectioning, the paraffin sections were stained with hematoxylin and eosin and examined using a light microscope.

### Statistical analysis

Statistical analysis of the results was carried out using Student’s *t*-test (*P* ≤ 0.05) (GraphPad Prism version 6). Comparison between the studied groups was carried out using *F*-test (ANOVA) and post hoc test (Newman–Keuls) for pair-wise comparisons. All statistical tests were two sided, and significance of the obtained results was judged at the 5% level.

## Results and discussion

### Preparation of fisetin–cholephytosomes

Generally speaking, phytosomes are phytochemical-phospholipid complex that can be developed at different molar ratios. In the current study, fisetin (FIS) was complexed for the first time with phospholipid in 1:1 molar ratio in attempt to improve its physicochemical properties and subsequently improve its therapeutic activities. Moreover, integration of cholesterol into the phytosomal system has not been so far employed in literature.

In order to develop cholesterol integrated fisetin phytosomes (FIS-cholephytosomes), fisetin and soy phosphatidylcholine (SPC) were initially dissolved in 1:1 molar ratio in a minimum amount of ethanol under continuous mild magnetic stirring at 400 rpm for 1 h at 40–45 °C to allow their complexation. Then, preweighed cholesterol was mixed with the previously developed 1:1 FIS/SPC complex just before injection into deionized water. Finally, the residual organic solvent was removed by vacuum evaporation technique. Three different cholephytosomal formulations were prepared with different cholesterol weights (Table 1). In order to select the optimum formulation, the prepared cholephytosomes were compared with conventional phytosomes (PLX) prepared through complexation of FIS and SPC in 1:1 molar ratio. The optimization of cholephytosomes were performed regarding their physicochemical properties in terms of particle size (PS), polydispersity (PDI), and surface charge.

**Table 1:** Physicochemical characterization of different cholesterol integrated -phytosomal dispersions (fisetin cholephytosomes)

**Formulation code**	**Cholesterol content (mg)**	**SA content (mg)**	**Particle size (mean ± SD)**	**PDI ± SD**	**Zeta potential (mV)**
**PLX**	0	-	242.67 ± 7.37	0.34 ± 0.09	− 29.47
**PHY1**	10	-	229.33 ± 3.06	0.30 ± 0.04	− 28.85
**PHY2**	15	**-**	226.67 ± 2.01	0.19 ± 0.02	− 28.06
**PHY3**	20	-	254.13 ± 9.12	0.33 ± 0.04	− 27.30
** + PHY1**	15	1	227.57 ± 4.21	0.37 ± 0.01	+ 35.21
** + PHY2**	15	5	236.67 ± 5.13	0.35 ± 0.04	+ 43.04
** + PHY3**	15	10	277.76 ± 25.58	0.44 ± 0.06	+ 51.43
mPHY^*^	15	5	248. 33 ± 4.86	0.30 ± 0.05	+ 41.40
HPHY *	15	5	277.66 ± 5.37	0.35 ± 0.04	− 21.73

*SA* stearylamine

1:1 FIS/SPC (PLX)

1:1 FIS/SPC with 10 mg cholesterol (PHY1)

1:1 FIS/SPC with 15 mg cholesterol (PHY2)

1:1 FIS/SPC with 20 mg cholesterol (PHY3)

cationic cholephytosomes (+ PHY)

modified cationic cholephytosomes with 1.25% w/w TPGS (mPHY)

*HPHY* optimized hyaluronic-coated Fisetin phytosomes

*Phytosomes with 1.25%w/v TPGS

As shown in Table 1, incorporation of cholesterol into the FIS-SPC dispersion could not cause dramatic change in the pharmaceutical properties of cholephytosomes. Compared with conventional phytosomes (PLX; 242.67 ± 7.37 nm), addition of moderate increments of cholesterol (10 and 15 mg) can increase the ordered arrangement of phospholipid membrane and its stability resulting in slight decrease in vesicle size to 229.33 and 226.67 nm, respectively [25, 46]. The size of vesicles increased to 254.13 nm when cholesterol concentration increased to 20 mg. According to the PDI values, all cholesterol-modified phytosomes show accepted PDI compared with conventional phytosomes (~ 0.3) confirming uniform and homogenous distribution of nanovesicles. Furthermore, zeta potential is also accounted for the prepared system stability. All FIS-cholephytosomes showed negative charge higher than − 25 mV, confirming their well-accepted colloidal stability. Among the tested formulations, fisetin cholephytosomes with 15 mg cholesterol (PHY2) showed smaller particle size with narrower PDI value (0.19 ± 0.02) along with stable negative surface charge (− 28.06 mV). That is why it was selected for further characterization.

### Preparation of active targeting fisetin PEGylated cholephytosomes

Fisetin cholephytosomes (PHY2) were furtherly modified in purpose of improving FIS delivery to breast cancer cells through either the externalized phosphatidylserine (PSe) or the overexpressed surface CD-44 receptors. First, cationic phosphatidylserine targeting cholephytosomes were developed using stearylamine (SA) as a positive charge inducer. SA was selected in the current work because it is reported to have specific and direct interaction with anionic surface exposed PSe better than other inducers such as N-[1-(2, 3-dioleoyloxy) propyl]-N,N,N-trimethylammonium chloride (DOTAP) and hexadecyltrimethylammonium bromide (CTAB) [47].

Initially, different concentrations of SA were tested to optimize the positive charge of nanovesicles. Cationic cholephytosomes containing 5 mg SA (+ PHY2) were selected for the decoration process (Table 1). It exhibited a stable positive surface charge (+ 43 mV) along with a non-significant increase in PS (237 ± 5.13 nm; *P* > 0.05) when compared with anionic cholephytosomes (PHY2; 226.67 ± 2.01 nm). Moreover, its surface charge remained nearly constant at + 41 mV for a week confirming the stability of such nanoformulation. It is worthy to mention that cholephytosomes containing 1 mg SA (+ PHY1) exhibited also promising results in terms of its particle size and zeta potential. However, it was excluded from the study because of instability of its surface charge. A drop in surface charge of freshly prepared sample was observed in the following 48 h (from + 35 mV to + 24 mV then + 18 mV after 24 h and 48 h, respectively).

The selected cationic cholephytosomes (+ PHY2) were furtherly stabilized with TPGS to produce PEGylated cationic cholephytosomes. TPGS is the synthetic water-soluble form of vitamin E. It is a bioactive non-ionic surfactant composed by the natural vitamin E bonded with polyethylene glycol 1000 (PEG). PEGylation of cationic cholephytosomes might reduce their non-specific interaction with blood components and their uptake by the reticuloendothelial system (RES), subsequently improving their tumor selectivity. Besides, it could be also utilized in cancer therapy as a P-glycoprotein efflux inhibitor reducing the multidrug resistance of cancer cells. Besides, the presence of vitamin E moiety allows TPGS to act as permeation enhancer and absorption enhancer [48, 49]. As demonstrated in Table 1, PEGylated cationic cholephytosomes containing 1.25% w/v TPGS (mPHY) showed a non-significant increase in PS (*P* > 0.05) and slight change in zeta potential (248.33 ± 4.86 nm, + 41.40 mV) compared with non-PEGylated cationic cholephytosomes (+ PHY2; 236.67 ± 5.13 nm, + 43.04 mV).

The CD-44 targeting cholephytosomes (HPHY) were then prepared through coating the surface of the PEGylated cationic cholephytosomes (mPHY) with hyaluronic acid (HA). Simple titration method was adapted to decorate the surface of modified cholephytosomes with the selected anionic polysaccharide. Concentrated stock solution of HA (5 mg/ml) was used to avoid unnecessary dilution of the sample. The titration method applied in this study is a non-washing coating technique exploited currently to avoid the multi-steps of washing and centrifugation related to the conventional technique [50]. Briefly, 1 ml of PEGylated cationic cholephytosomes (mPHY) was titrated with different volumes of concentrated stock of HA solution (0.1–0.5 ml). The optimum volume will be the minimum volume that could inverse the positive charge into a stable and acceptable negative charge beside avoiding the presence of excess electrolytes to maintain uniform coating and quality of developed nanovesicles [51].

**Fig. 1:**
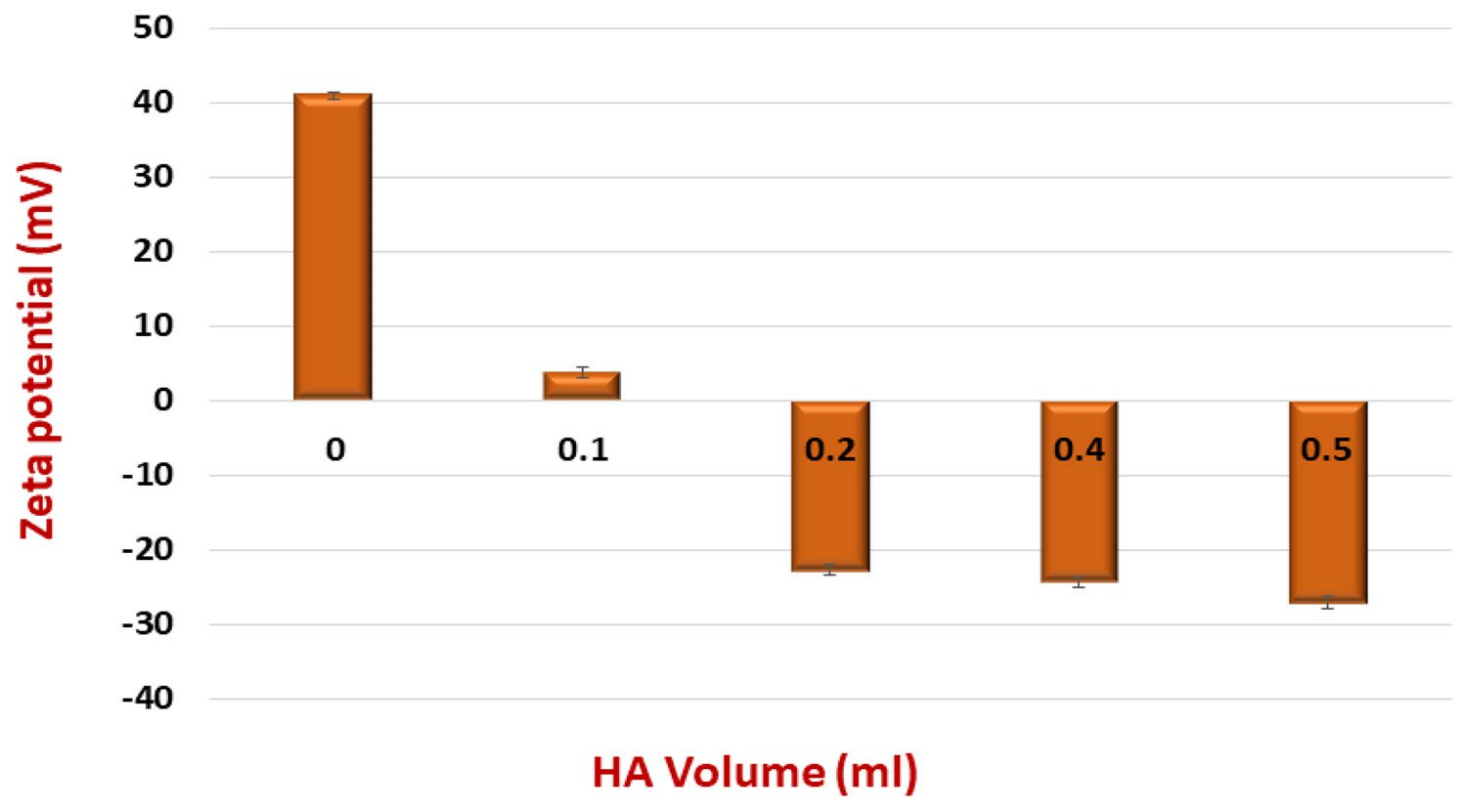
Effect of the gradual titration of anionic biopolymer (hyaluronic acid; HA) on zeta potential of optimized cationic FIS-PEGylated cholephytosomes (mPHY)

As shown in Fig. 1, the negative charge started to appear on surface of nanovesicles using 0.2 ml of HA solution. Upon increasing HA volume, slight increase in the zeta potential was detected. Therefore, the optimum volume for HA solution was selected to be 0.2 ml. The size and PDI of were measured to exploit the effect of coating on other physical properties. Regarding particle size, HA-coated cholephytosomes (HPHY) showed an increase in particle size (277.67 ± 5.37 nm) compared with uncoated cationic cholephytosomes (+ PHY2; *P* < 0.05). Such increase in PS after coating process confirmed the adsorption of HA molecules (MW 100 KDa) on cationic surface of cholephytosomes [52, 53].

### Complexation efficiency (CE %)

Complexation efficiency was measured to modified cholephytosomes (mPHY and HPHY) in comparison with conventional drug-phospholipid complex (PLX) in order to investigate the influence of structural modification of complex on fisetin interaction with phospholipid. At 1:1 molar ratio, complexation of fisetin with phospholipid successfully reached to 100%. It is worthy to mention that addition of cholesterol to liposomes was reported to reduce entrapment efficiency of some flavonoids such as curcumin [54]. Meanwhile in the current study, modification of FIS/SPC complex with cholesterol and other excipients did not affect interaction between fisetin and phospholipid where complexation efficiency remained almost constant at 100%. This could confirm the strong interaction and bonding between fisetin and the phospholipid where the formed complex has not been even partially dissociated upon modification employed to the complexed system.

### Octanol–water solubility study

The solubility of free fisetin in deionized water and n-octanol was determined and compared with the Chol/SA/TPGS-modified FIS-SPC complex. As presented in Table 2, pure FIS showed poor aqueous solubility (2.52 ± 0.15 mg%), with higher solubility in n-octanol (191.18 ± 1.41 mg%) confirming its lipophilic nature. When fisetin was complexed with SPC, a significant increase in its aqueous solubility (3.36-fold, *P* < 0.01) and in n-octanol (4-folds, *P* < 0.001) was observed compared to that of the free FIS. The enhanced aqueous and oil solubility of fisetin phospholipid complex can be attributed to the amphiphilic and amorphous nature of the formed complex [55, 56]. Moreover, incorporation of surfactant such as TPGS could also contribute to the overall improvement in the aqueous solubility of complexed FIS.

**Table 2:** Solubility results of pure FIS and modified FIS–SPC in n-octanol and in water

**Sample**	Solubility in DI (mg%) *	**Solubility in n-octanol (mg%)**
**Pure fisetin**	2.52 ± 0.15	191.18 ± 1.41
**Modified FIS-SPC**	8.46 ± 0.23	781.02 ± 3.47

*Results are expressed in term of mean ± SD (*n* = 3)

*DI* deionized water.

### Fourier transform infrared spectroscopy (FTIR)

FTIR study was furtherly employed to investigate the possible interaction between FIS and SPC responsible of complex formation (Fig. S1). Compared with physical mixture spectrum, the spectra of conventional FIS-phytosomes (D) and the modified cholephytosomes (E and F) showed marked spectroscopic changes in the characteristic peaks of their components (refer to supplementary for detailed description).

### Transmission electron microscopy (TEM)

**Fig. 2:**
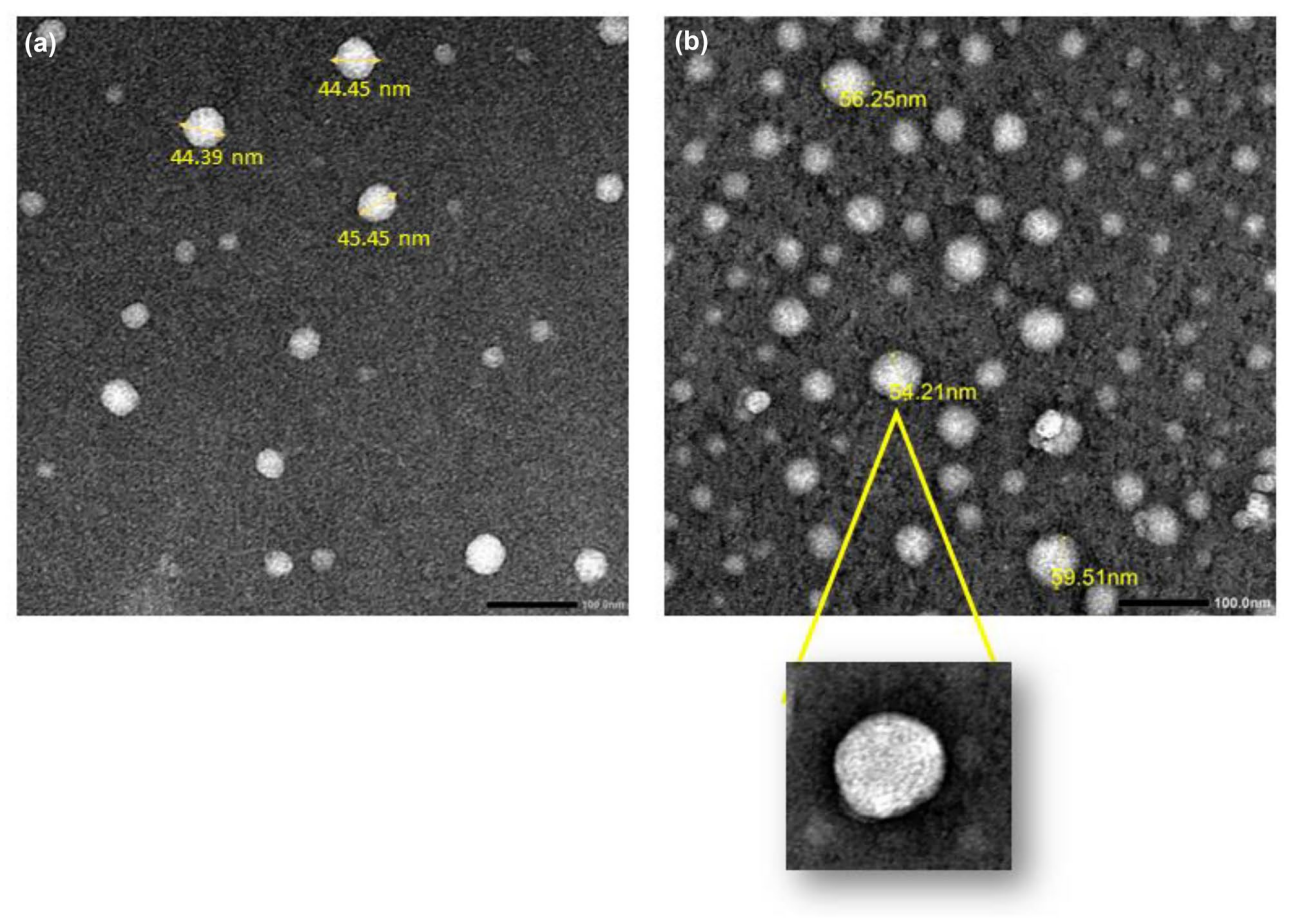
TEM photomicrographs of mPHY (**a**) and HPHY (**b**) at magnification = 50,000 × . Scale bar = 100 nm

**Fig. 3:**
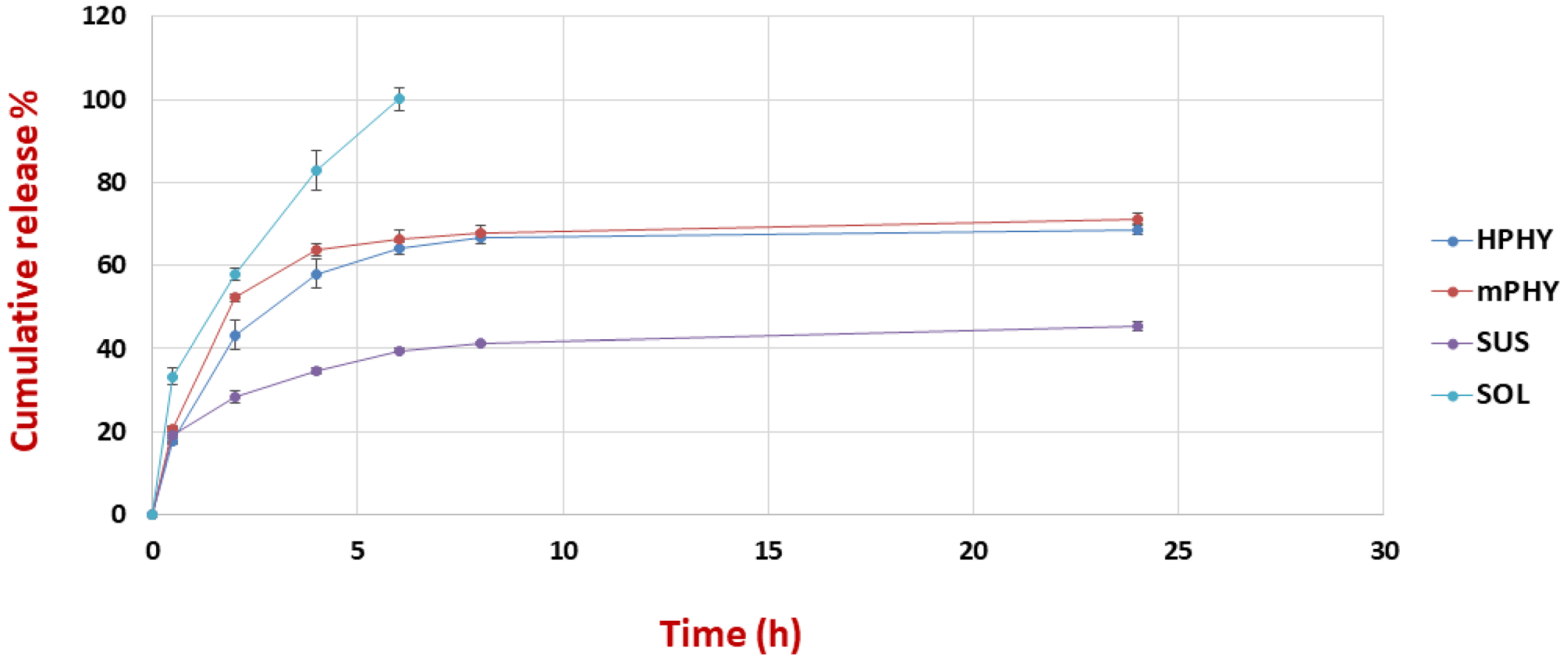
In vitro release profiles of free FIS solution (SOL), FIS suspension (SUS), cationic FIS-PHY (mPHY), and HA-coated phytosomes (HPHY) using dialysis method in phosphate-buffer saline (PBS) pH 7.4 containing 20% alcohol at 100 rpm and 37 °C. Results are expressed as mean ± SD (*n* = 3); some error bars are too small to be presented

As shown in Fig. 2, modified cationic fisetin nanovesicles (mPHY) and hyaluronic decorated nanovesicles (HPHY) exhibited uniform vesicular forms that are almost spherical in their shape with no sign of aggregation. Similar to results of particle size analysis via DLS, the coating of cationic cholephytosomes (mPHY; Fig. 3) could be confirmed by TEM through increasing the size of polysaccharide coated cholephytosomes (HPHY; Fig. 3). However, the size of tested nanovesicles appeared smaller than that measured by DLS. This could be ascertained to the dehydration step carried out during TEM processing which may shrink the vesicles. Furthermore, the dark outer layer around targeted nanovesicles could also confirm the successful coating of cationic nanovesicles with hyaluronic acid (HA) [36].

### In vitro release study

The in vitro release of fisetin from the modified cholephytosomes (mPHY and HPHY) was determined over 24 h using standard dialysis bag. As shown in Fig. 3, complexation of fisetin with the amphiphilic phospholipid could improve its lipophilicity. This was clearly obvious through its sustained release from cholephytosomes unlike its fast release from its solution (*P* < 0.01) which exhibited about 58% release after 2 h and reached to 100% release after 6 h. Furthermore, the aqueous solubility of complexed FIS was also improved where its release pattern was significantly enhanced in terms of its extent and rate when compared with its suspension which exhibited 45.30% over 24 h (*P* < 0.01). It is worthy to mention that crude fisetin showed to some extent good aqueous solubility in the release medium which could be attributed to the partial ionization of fisetin molecules at pH 7.4 of the release medium. According to structure of fisetin, it has four acid–base dissociation constants (*pKa* 7.3 for C7-OH, 8.5 for C4′-OH, 12.2 for C3-OH, and 13.8 for C3′-OH) [40]. Therefore, it is expected that at pH 7.4 of release medium, the -OH group at the C-7 position would be ionized (*pKa* 7.3) and there would be an equilibrium between the ionized fisetin molecules and their unionized form. Consequently, the ionized fisetin would diffuse easily into the aqueous medium enhancing the overall release pattern of fisetin suspension.

Fisetin release from tested nanoformulations showed biphasic release pattern. In the first 6 h, the drug revealed an initial burst release of 64.15% and 66.28% from HPHY and mPHY, respectively, followed by sustained release for 24 h. The biphasic release pattern of fisetin from the optimized cholephytosomes might be due to the fast release of ionized fisetin molecules at pH 7.4 (*pKa* 7.3 for C7-OH). Then a sustained release of the non-ionized complexed drug was dominated by its gradual dissociation from the phospholipid complex followed by its subsequent diffusion through dialysis bag into the release medium. Eventually, coating cholephytosomes with hyaluronic acid could slightly retain FIS release in the first 6 h from HPHY when compared with mPHY. The hydrophilic hyaluronic acid coat could form hydrophilic channels upon swelling allowing diffusion of FIS easily into the release medium. This could explain why there was no significant difference in the release pattern of fisetin form both hyaluronic-coated and cationic cholephytosomes (*P* > 0.05).

### Hemocompatibility test

Both surface modified cholephytosomes (mPHY and HPHY) demonstrated hemolytic activity below the accepted limit (5%). Compared with HA-coated cholephytosomes (HPHY) which showed non-detectable and almost neglectable blood hemolysis (0.50 ± 0.14%), stearylamine modified cationic cholephytosomes (mPHY) exhibited around 3.35 ± 0.27% hemolysis (Fig. S2). These results confirmed that coating the positively charged cholephytosomes with hydrophilic biopolymer such as hyaluronic provided a protective barrier in the outer layer of fisetin cholephytosomes and subsequently inhibited the interaction of such vesicles with plasma components. This would result in improving their biocompatibility, in vitro and in vivo stability.

### Short shelf-life storage stability of lyophilized fisetin cholephytosomes

Removal of water from the pharmaceutical formulations is a critical step in pharmaceutical industry in order to improve the final product stability and its shelf-life [57]. Therefore, lyophilization was carried out in the current study to obtain the optimized cholephytosomes in powder form. In addition, short-term shelf-life stability study was conducted to investigate the physicochemical properties of the dried cholephytosomes in terms of their particle size, zeta potential, and also entrapment efficiency.

Preliminary investigations revealed that trehalose was the cryoprotectant of choice for the lyophilization process. Initially, reconstitution of all tested lyophilized cholephytosomes prepared without addition of lyoprotectant resulted in poly dispersed vesicles with significant increase in their particle size. Therefore, both mannitol and trehalose at 2% w/v were tested for preparing lyophilized samples with proper physicochemical properties. Compared with their corresponding freshly prepared nanoformulations, it was observed that addition of 2% (w/v) mannitol resulted in poly dispersed vesicles (PDI > 0.3) upon reconstitution along with significant increase in their particle size (*P* < 0.05) as illustrated in Table S1. This could be attributed to the crystallization of mannitol which could increase the mechanical forces on the cholephytosomes leading to their aggregation [58] On other hand, the physicochemical characteristics of all lyophilized cholephytosomes did not change significantly in the presence of 2% (w/v) trehalose upon reconstitution with deionized water and even after 4 weeks of storage in desiccator at room temperature (Table 3). This in turn could reflect their promising stability. However, further complete stability studies should be done for full assessment of fisetin cholephytosomes stability.

**Table 3:** Short shelf-life stability of freeze-dried fisetin cholephytosomes

Parameter	Fresh prepared cholephytosomes		Freeze-dried cholephytosomes*			
			**(At zero time after lyophilization)**		**(After 4 weeks of lyophilization)**	
	**mPHY**	**HPHY**	**mPHY**	**HPHY**	**mPHY**	**HPHY**
**PS (nm)**	246.52 ± 2.61	275.26 ± 3.74	250.98 ± 5.10	281.36 ± 3.90	254.23 ± 5.37	280.04 ± 6.22
**PDI**	0.32 ± 0.02	0.29 ± 0.05	0.34 ± 0.07	0.24 ± 0.06	0.37 ± 0.07	0.35 ± 0.07
**ZP (Mv)**	+ 42.07 ± 1.84	–22.41 ± 1.05	+ 39.45 ± 2.09	-20.26 ± 2.18	+ 38.01 ± 1.78	-20.90 ± 2.04
**EE %**	98.05 ± 0.48	98.58 ± 0.53	98.11 ± 0.41	98.10 ± 0.51	97.18 ± 0.24	97.52 ± 0.16

*Lyophilized in the presence of 2% (w/v) trehalose and stored in desiccators at room temperatures

*mPHY* modified cationic fisetin cholephytosomes, *HPHY* hyaluronic decorated fisetin cholephytosomes, *PS* particle size, *PDI* poly dispersity index, *ZP* zeta potential, *EE %* entrapment efficiency

*Results of particle size measured as mean* ± *SD (*n = 3)

### In vitrocell line studies

MDA-MB-231, a human triple negative breast cancer (TNBC) cell line, was selected for the current in vitro cell line studies as it is reported to overexpress CD-44 receptors [59, 60] and also expose PSe on their surface [61, 62]. This would facilitate studying the targeting potential of both HA-decorated fisetin cholephytosomes (HPHY) and SA-bearing cationic fisetin cholephytosomes, respectively, (mPHY) and comparing their antitumor efficacy as well.

#### Cellular cytotoxicity assay (MTT assay)

The cytotoxicity of mPHY and HPHY on MDA-MB-231 cells was assessed using MTT assay. Free FIS and conventional phytosomes (PHY) were also appraised to study the effect of FIS complexation with SPC and also modification of phytosomes on FIS anticancer activity compared with modified targeted cholephytosomes. Different concentrations of the selected formulations were tested to determine their IC 50 (half maximal inhibitory concentration). Then, IC50 results were used to compare the antitumor activity of different fisetin nanovesicles against cancerous cell line and also to determine the appropriate concentration required for subsequent in vitro cell line studies.

**Fig. 4:**
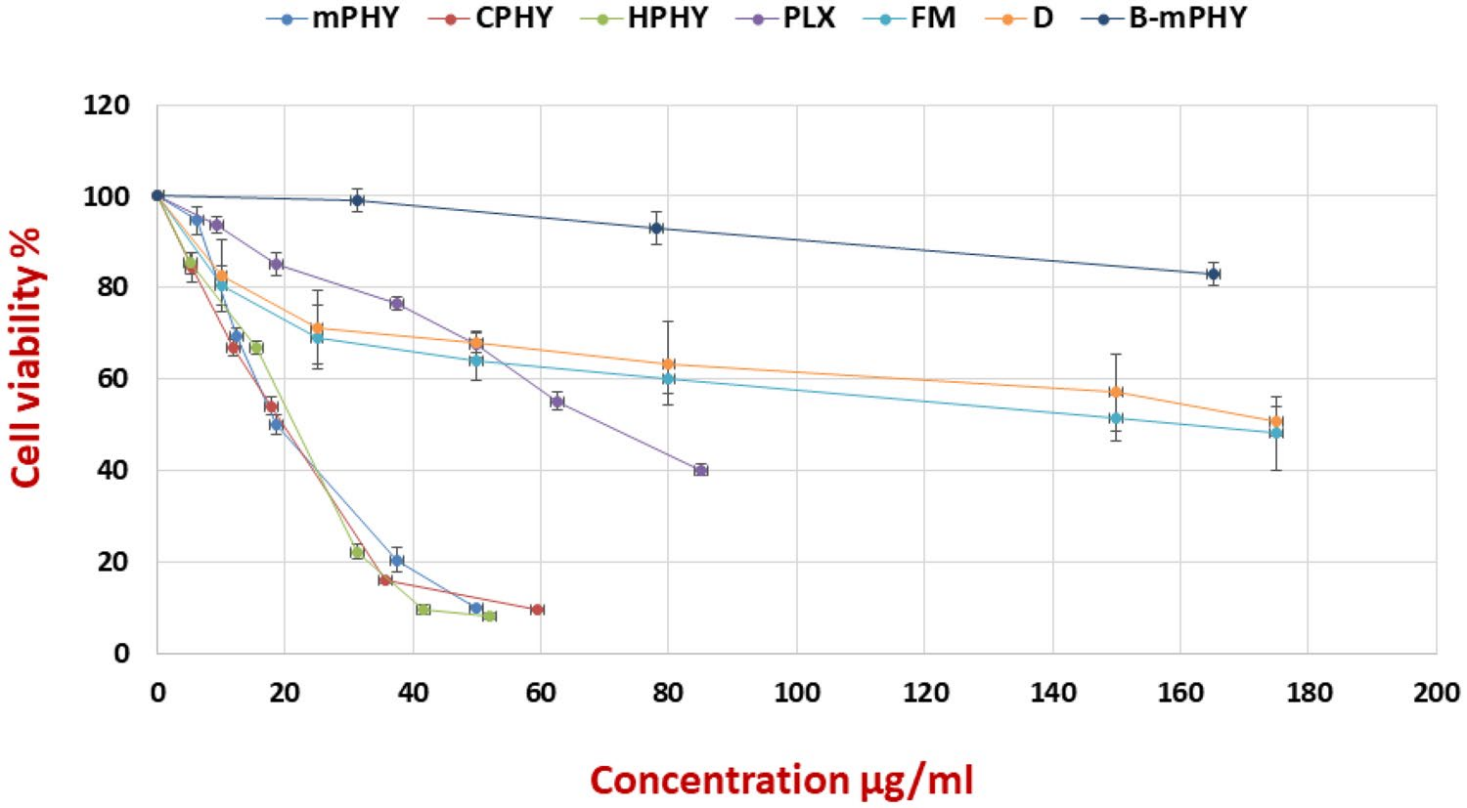
Viability assay of free fisetin (D), physical mixture of FIS and SPC (FM), conventional FIS-phytosomes (PLX), cationic fisetin cholephytosomes (mPHY), blank cationic nanovesicles (B-mPHY), and HA-coated FIS-cholephytosomes (HPHY) after incubation for 24 h with MDA-MB-231 breast cancer cells

**Fig. 5:**
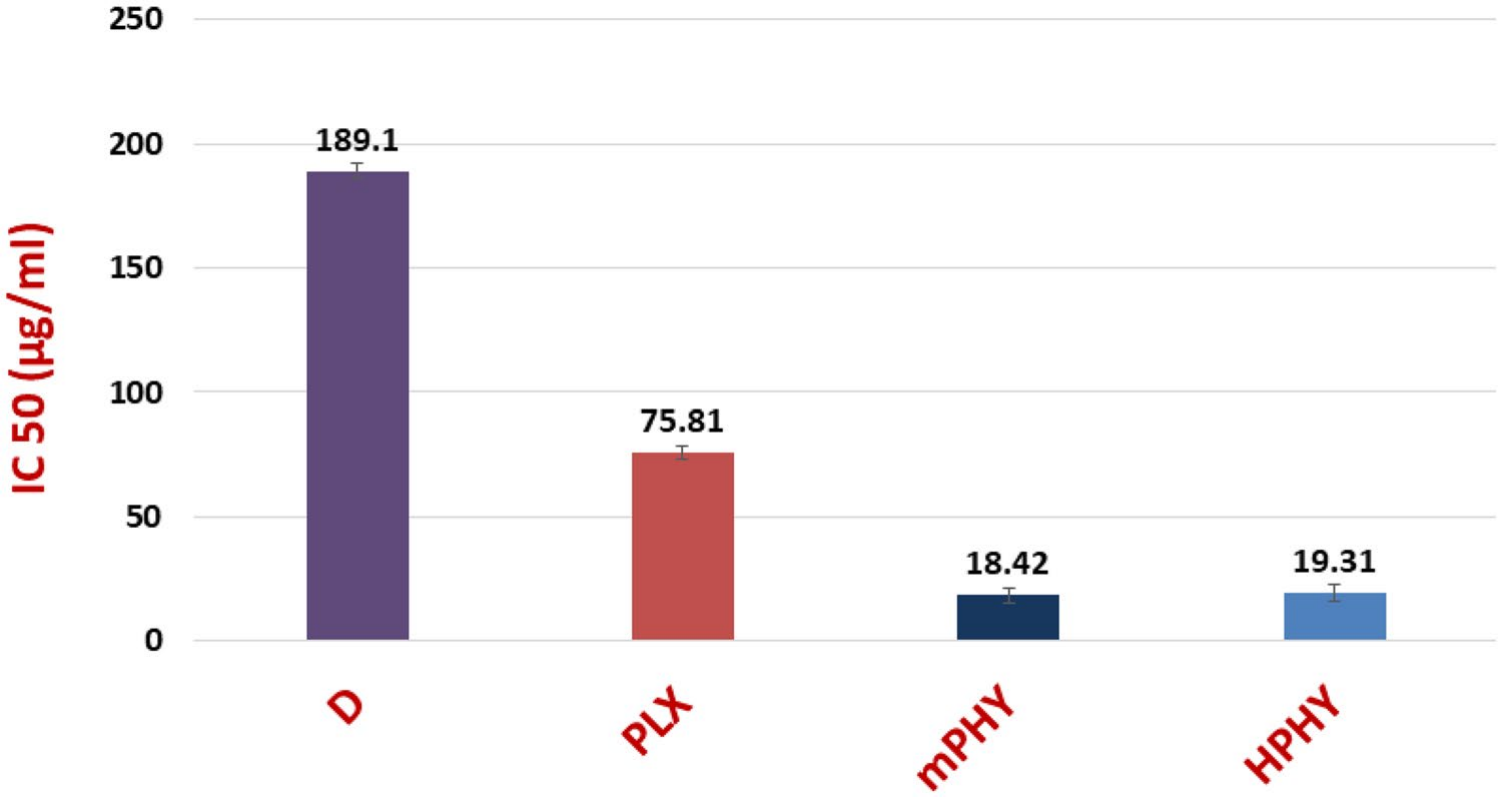
IC50 in µg/ml for free FIS (D) and different FIS-phospholipid complexes against MDA-MB-231 breast cancer cell line after 24 h. Conventional FIS-phytosomes (PLX), cationic fisetin cholephytosomes (mPHY), and HA-coated FIS-cholephytosomes (HPHY)

As shown in Fig. 4, fisetin exhibited concentration-dependent cytotoxicity from all tested formulations. Furthermore, the surface modifications of conventional fisetin phytosomes with SA and HA (mPHY and HPHY) showed outstanding impact on fisetin cytotoxicity. Compared with conventional FIS-phospholipid complex (PLX; IC50 of 75.81 ± 2.99 µg/ml), SA-bearing cholephytosomes (mPHY) and HA-coated cholephytosomes (HPHY) showed 4.12-fold and 3.93-fold higher cytotoxicity (IC50 of 18.42 ± 2.90 µg/ml, 19.31 ± 3.56 µg/ml), respectively, (*P* < 0.001, Fig. 5).

The significant enhancement of fisetin cytotoxicity observed with modified cholephytosomes can be imputed to different factors. First, modification of nanovesicles with selective targeting ligands could increase fisetin internalization into cancer cells. In case of HPHY, enhanced FIS uptake occurred through CD-44 receptors mediated endocytosis. While in case of mPHY, stearylamine (SA) can fuse selectively with the negatively charged phospholipid (PSe) exposed on the cellular membrane of cancer cells due to its positive charge resulting in enhanced cellular uptake and internalization of nanoparticles [47, 63]. Second, TPGS which was used as stabilizer for modified cholephytosomes is a multifunctional surfactant possessing antitumor activity that could synergize the antitumor activity of fisetin [64]. In addition, TPGS can also promote internalization of fisetin and limit its efflux outside cancer cells [65].

It is worthy to mention that despite its tumor permeability enhancing role, SA is known to have toxicity by itself on cells at certain concentrations. Thus, it should be added to nanoparticles with appropriate concentration to ensure their systemic safety and avoid interaction with biological membranes as RBCs. Accordingly, fisetin-free nanovesicles (blank cationic nanovesicles; B-mPHY) were prepared and applied to cancer cells in different concentrations of SA to investigate if the significant enhancement of fisetin cytotoxicity was due to the enhanced drug internalization into cancer cells or due to SA own toxicity. Generally, cell viability above 70% is considered nontoxic, while less than 50% is considered to be cytotoxic [66]. The results of cell viability assay (Fig. 4) confirmed the safety of blank cationic nanovesicles where at a concentration equivalent to IC 50 of cationic fisetin cholephytosomes (18.42 µg/ml), the blank cationic formulation which contained SA of 6.25 µg/ml showed cellular viability of 99%. Even higher concentrations of SA reaching to 31.25 µg/ml showed up to 83% cell viability. Such results confirmed that cationic fisetin cholephytosomes exhibited their enhanced cytotoxicity due to improving fisetin internalization not due to the toxic effect of SA. A final concentration which was approximating IC50 of fisetin cholephytosomes (20 µg/ml) was appropriate to be applied in the further cell line studies.

#### Apoptosis assay (annexin-V-FITC/propidium iodide assay)

**Fig. 6:**
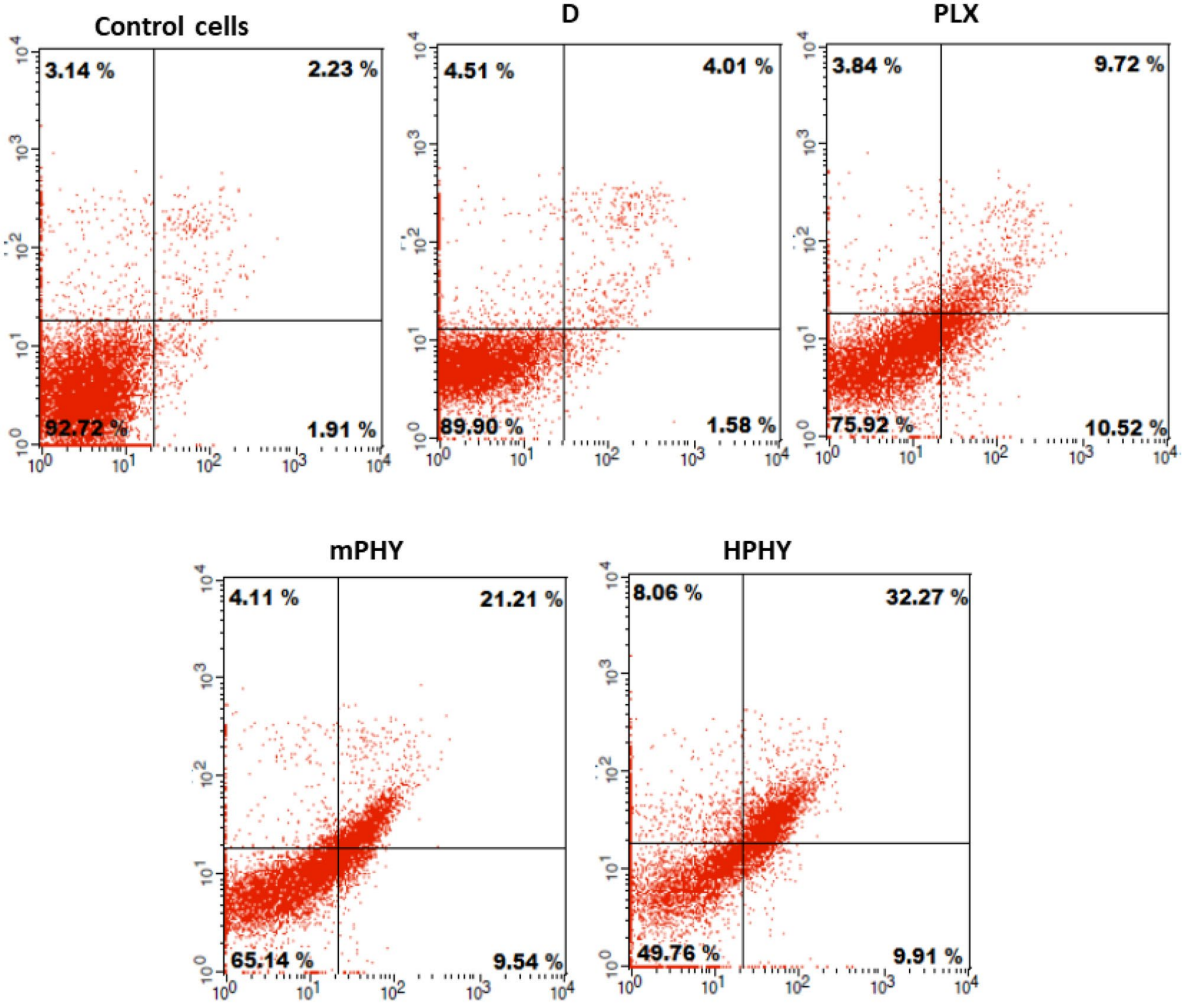
Apoptotic assay for FIS-phytosomes against fisetin solution at 20 µg/ml equivalent dose of FIS using annexin-v-FITC/propidium iodide assay by flow cytometry after incubation for 24 h with MDA-MB-231 cells. Abbreviations; D fisetin solution in DMSO, PLX conventional FIS-phytosomes, mPHY cationic FIS-cholephytosomes, HPHY hyaluronic-coated FIS-cholephytosomes

Annexin V/propidium iodide assay was performed to assess the cytotoxicity mechanism of fisetin after treatment cells with free drug and nanoformulations (20 µg/ml fisetin) for 24 h (Fig. 6). The flow cytometry diagrams confirmed that free fisetin and its complexed forms induced cytotoxicity in MDA-MB-231 cells mainly through apoptosis.

Compared with Free FIS, complexation with SPC (PHY) resulted in a significant enhancement of FIS cytotoxicity (*P* < 0.01) as demonstrated in Fig. 6. Similar to results of MTT assay, superior cytotoxicity of fisetin against cancer cells was achieved upon modification of conventional phytosomes (PHY) with targeting ligands (SA and HA). Both cationic SA-bearing PEGylated cholephytosomes (mPHY) and HA-coated PEGylated cholephytosomes (HPHY) showed 1.45-fold and 2.1-fold higher total death percentage than conventional phytosomes (*P* < 0.001) by the end of 24-h assay. In addition, the apoptosis assay revealed significant higher total cell death of ~ 50% accounted for HA-coated PEGylated cholephytosomes (HPHY) after 24 h when compared with cationic PEGylated cholephytosomes (mPHY) that showed a total cell death of ~ 35% (*P* < 0.01).

#### Nuclear morphological assay (confocal microscopic study)

**Fig. 7:**
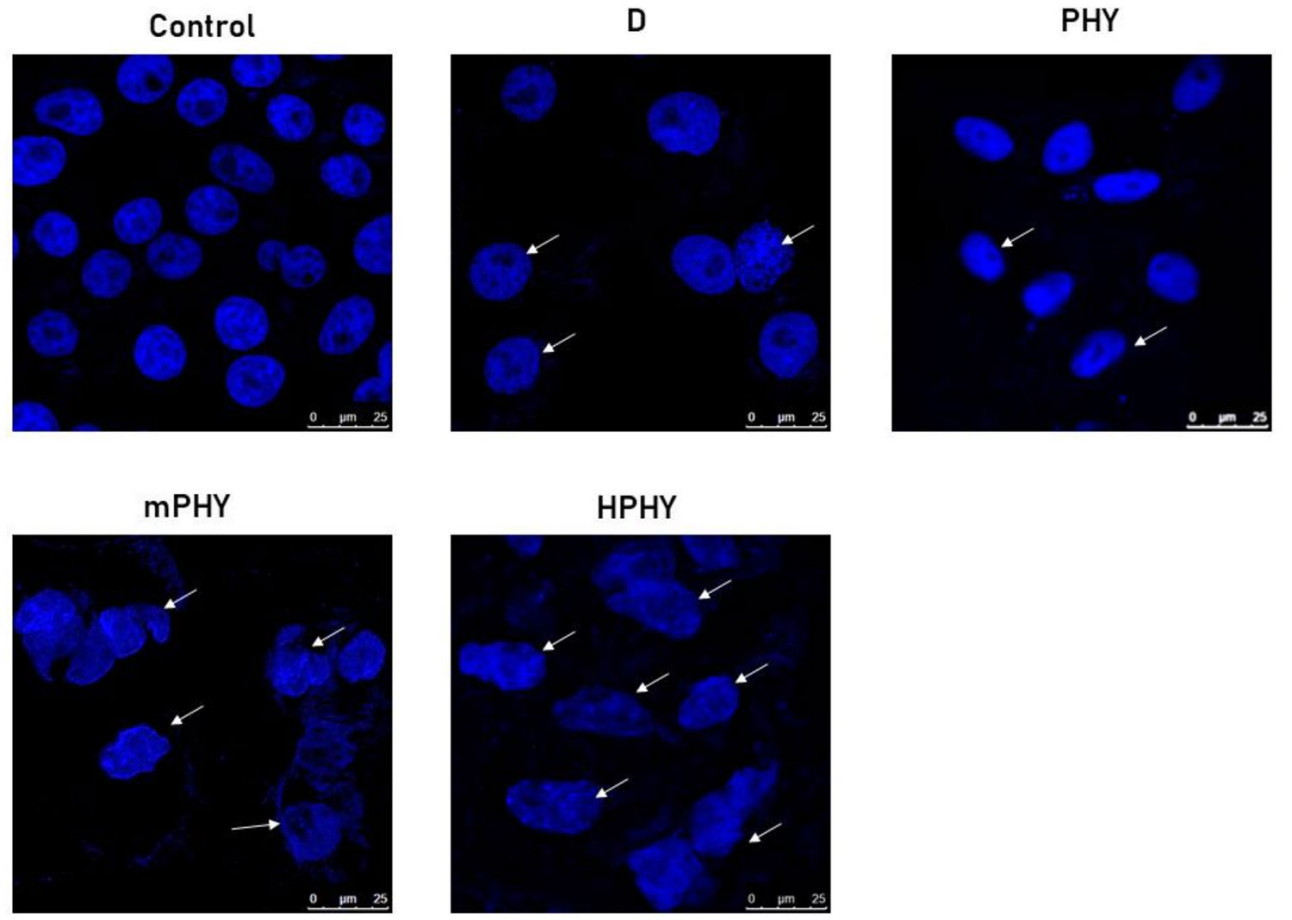
Assessment of nuclear morphology by DAPI staining of MDA-MB-231 nuclei using confocal microscope. Photographs of control cells showed normal and uniform nuclear structure with a large and round nucleus and uniform chromatin density. Treated cells with 20 µg/ml of fisetin (F) or equivalent dose of conventional phytosomes (PLX) and modified cholephytosomes (mPHY and HPHY) showed a significant change in nuclear morphology. White arrows manifest fragmented nuclei with chromatin condensation. Incubation time was 24 h, and the scale bar is 25 µm

The quantitative apoptosis analysis was further supplemented by the qualitative assessment of the nuclear morphology using confocal microscope and DAPI staining. As shown in Fig. 7, the nuclei of control group showed homogenous blue fluorescence with round cell nuclei and the absence of any fragmentation. While clear morphological changes were manifested including nuclei shrinkage, chromatin condensation and DNA fragmentation when the cells were treated with FIS or other fisetin nanovesicles. Such changes were observed after treatment for 24 h with 20 µg/ml of FIS or to its equivalent dose from nanoformulations (20 µg/ml)**.** The presence of fragmented nuclei was obviously greater and abundant for both cationic and HA-coated FIS-pegylated cholephytosomes (mPHY and HPHY) compared to pure FIS and conventional phytosomes (PHY). It is worthy to note that these results were in accordance with the quantitative apoptosis assay where fisetin in its complexed form either as conventional phytosomes or modified cholephytosomes showed higher percentage of apoptosis relative to free fisetin and control.

Overall, in vitro cell line studies against MDA-MB-231 cells revealed significant superior anticancer efficacy of the modified cholephytosomes (mPHY and HPHY) over conventional phytosomes (PHY) and free uncomplexed FIS. Therefore, comparative in vivo studies were conducted on modified cholephytosomes (mPHY and HPHY) to furtherly confirm their antitumor efficacy on breast cancer induced animal model.

### In vivo anti tumor activity studies

In the current preclinical studies, subcutaneous Ehrlich Ascites carcinoma (Ehrlich cells, EAC) was employed as a well-established tumor model for breast cancer [67]. It is worthy to mention that both CD-44 receptors [68, 69] and surface exposed Pse [70, 71] have been successfully utilized in different in vivo breast cancer models to target various anticancer drugs to cancerous cells.

#### Effect of modified cholephytosomes on tumor growth rate

**Fig. 8:**
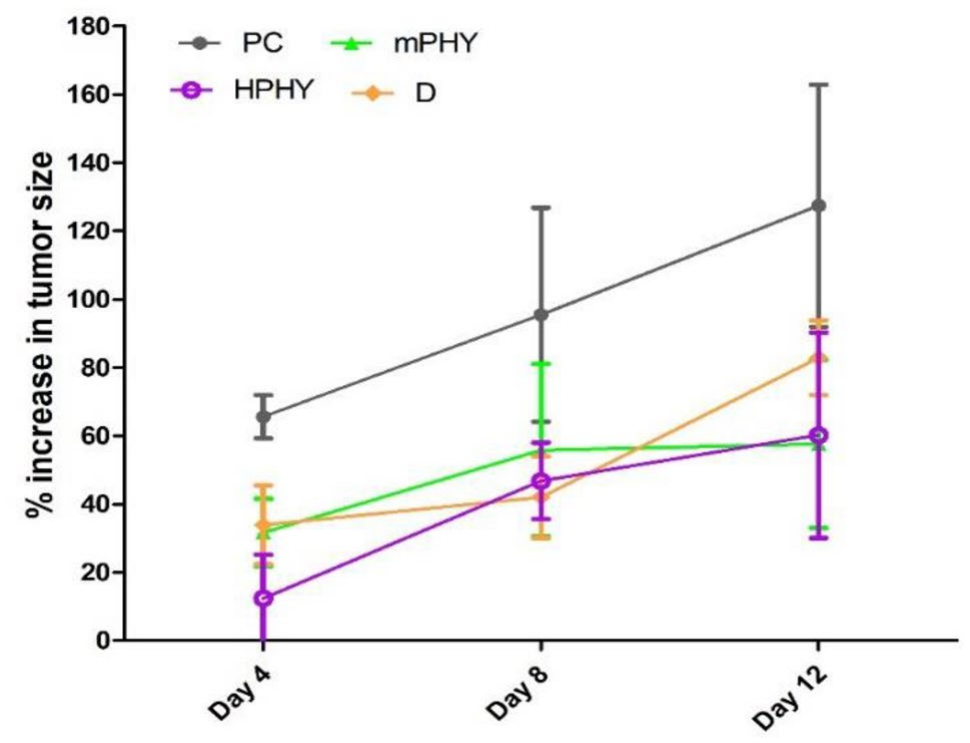
Effect of FIS (D) and modified cholephytosomes (mPHY and HPHY) on percent increase of tumor size. PC untreated mice, D FIS solution, mPHY cationic SA-bearing cholephytosomes, and HPHY hyaluronic decorated cholephytosomes. Two-way ANOVA was used to test effect of treatment and followed by Bonferroni test

After 12 days of treatment, the tumor growth rate in the untreated mice group (PC) showed 128% increase in size, while treatment with FIS solution (D) exhibited slight improvement representing around 85% increase. On the other hand, groups treated with modified cholephytosomes (mPHY and HPHY) could pronouncedly affect tumor growth rate where both of them revealed a comparable effect causing about 50% increase on day 12. It is noteworthy that free FIS treated group (D) has recorded approximately double increase in tumor size (from 42 to 85%) from days 8 to 12. On the other hand, only 3% and 10% increase were observed at the same period upon treatment with mPHY and HPHY, respectively. Two-way ANOVA showed a significant effect of time (df = 5.31) and treatment (df = 3.66). Results are shown in (Fig. 8).

#### Effect of FIS-cholephytosomes on TGF-β pathway induced tumorigenesis

Subcutaneous EAC have been reported to have a potential for invasion and also metastasis to the lungs, bone, liver, spleen, and kidney [67]. In fact, the TGF-β1 signaling pathway has been proven to play a pivotal role in tumorigenesis, cancer progression, and metastasis [72].

**Fig. 9:**
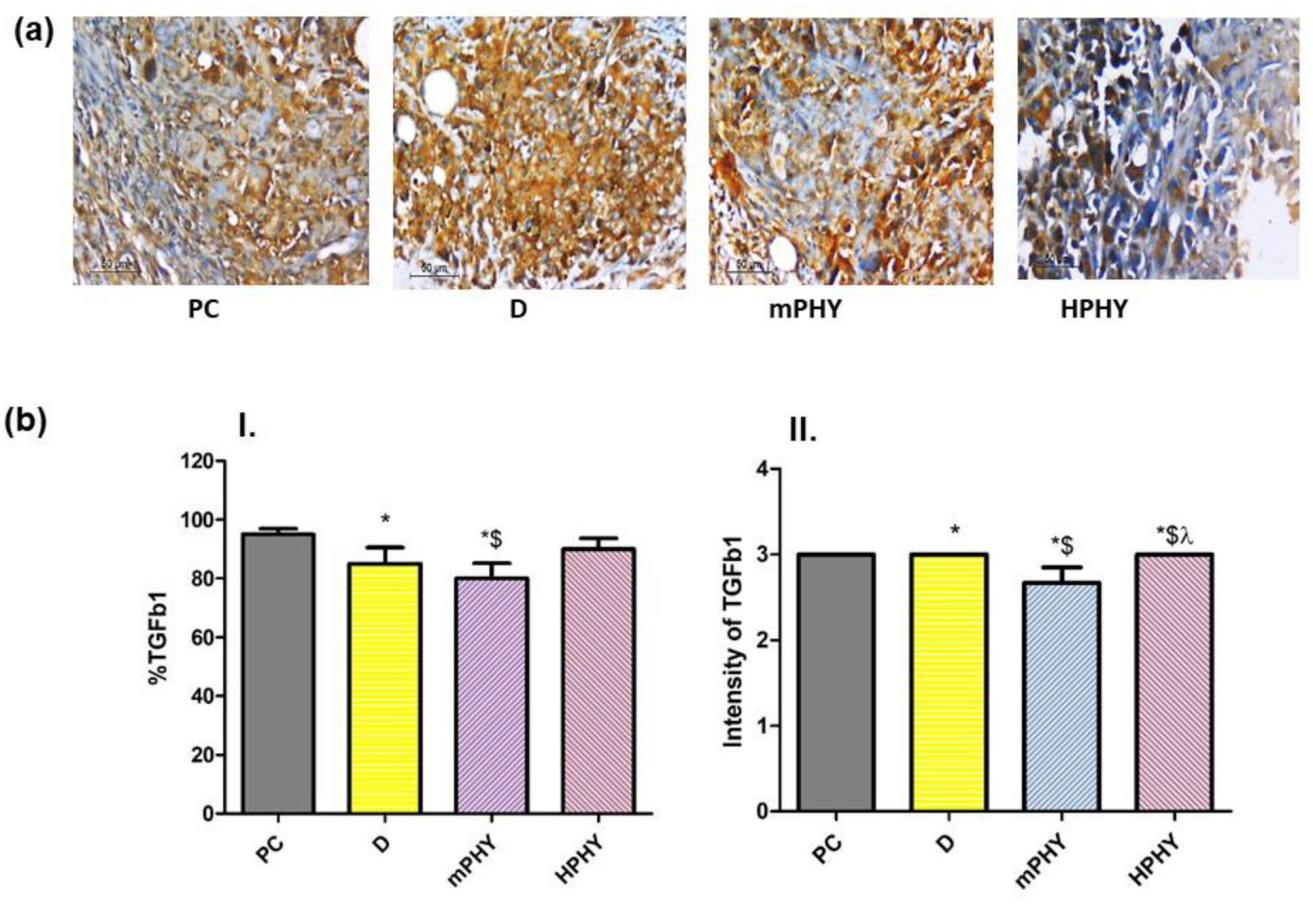
Effect of FIS (D) and modified cholephytosomes (mPHY and HPHY) on immunohistochemical expression of TGB-1 in breast carcinoma: representative photomicrograph (TGB-1, X400) (**a)** and quantitative determination (**b**) of (I) percent immunostaining and (II) intensity of stain. PC: untreated mice, mPHY: cationic SA-bearing cholephytosomes, HPHY: hyaluronic decorated cholephytosomes. Statistical analysis was done using one-way ANOVA followed by Student–Newman–Keuls multiple comparison test; *P < 0.05 vs PC, $*P* < 0.05 vs D, *λ P* < 0.05 vs mPHY

**Fig. 10:**
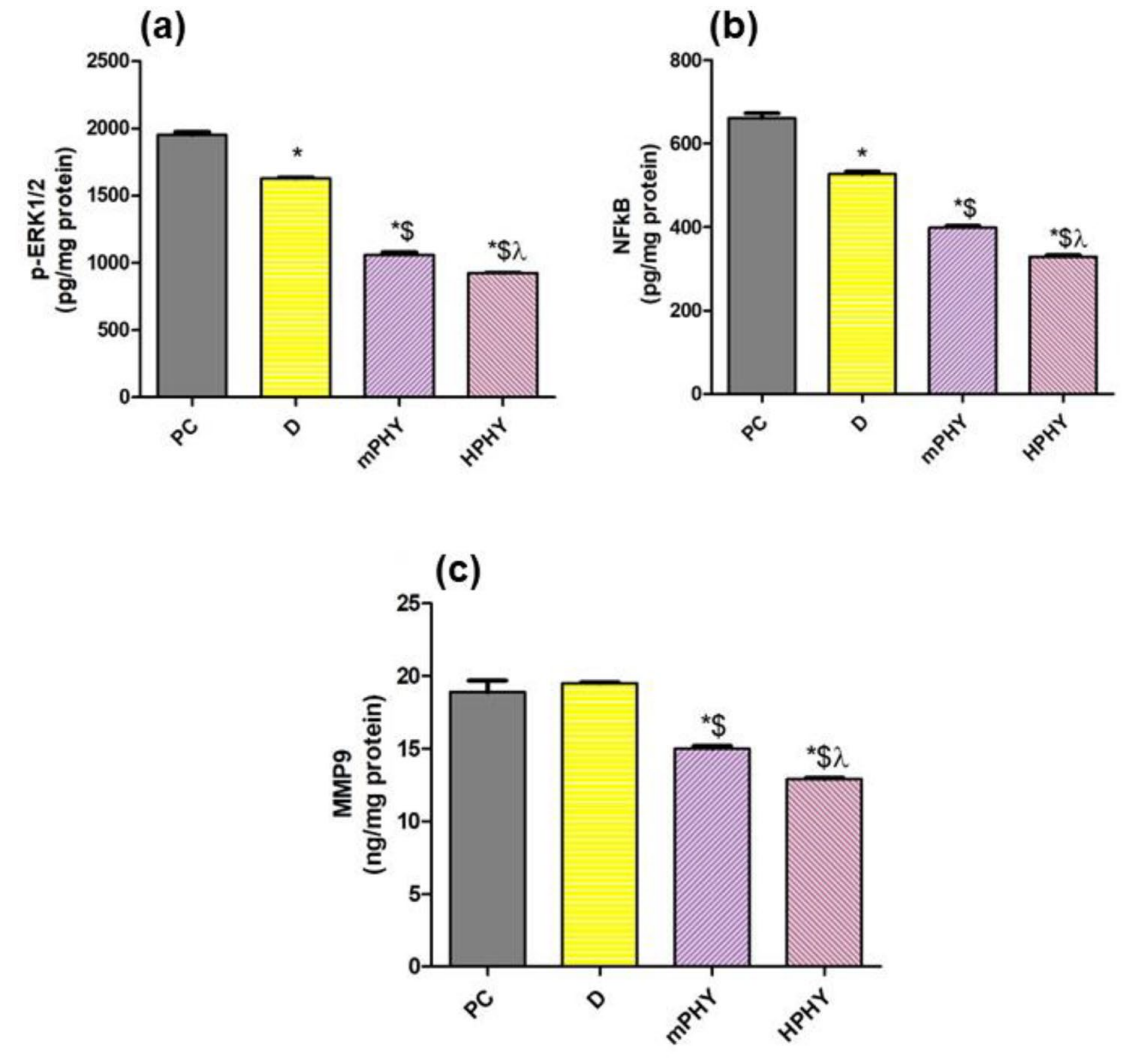
FIS (D) and modified cholephytosomes (mPHY and HPHY) on: p-ERK1/2 (**a**), NF-κb (**b**), MMP9 (**c**). PC untreated mice, mPHY cationic SA-bearing cholephytosomes, and HPHY hyaluronic decorated cholephytosomes. Statistical analysis was done using one-way ANOVA followed by Student–Newman–Keuls multiple comparison test; *P < 0.05 vs PC, $*P* < 0.05 vs D, *λ P* < 0.05 vs mPHY

As shown in Fig. 9, the untreated breast cancer cells (PC) have shown increased TGF-β1 content upon immunological staining. Such elevation resulted in activation of non-SMAD pathways including an increased MMP-9, ERK1/2, and NF-κB levels (Fig. 10). The ability of TGF-β1 to activate non-canonical related pathways has been previously proven in many contexts interacting with several transcription factors to regulate gene expression [73]. Wendt et al. [74] and others [75, 76] have shown that TGF-β enhanced breast cancer invasion by inducing MMP-2 and MMP-9 expression. Such modulation of MMPs expression has shown to be mediated through ERK1/2 and NF-κB signaling pathways in metastatic cancer [77, 78].

**Fig. 11:**
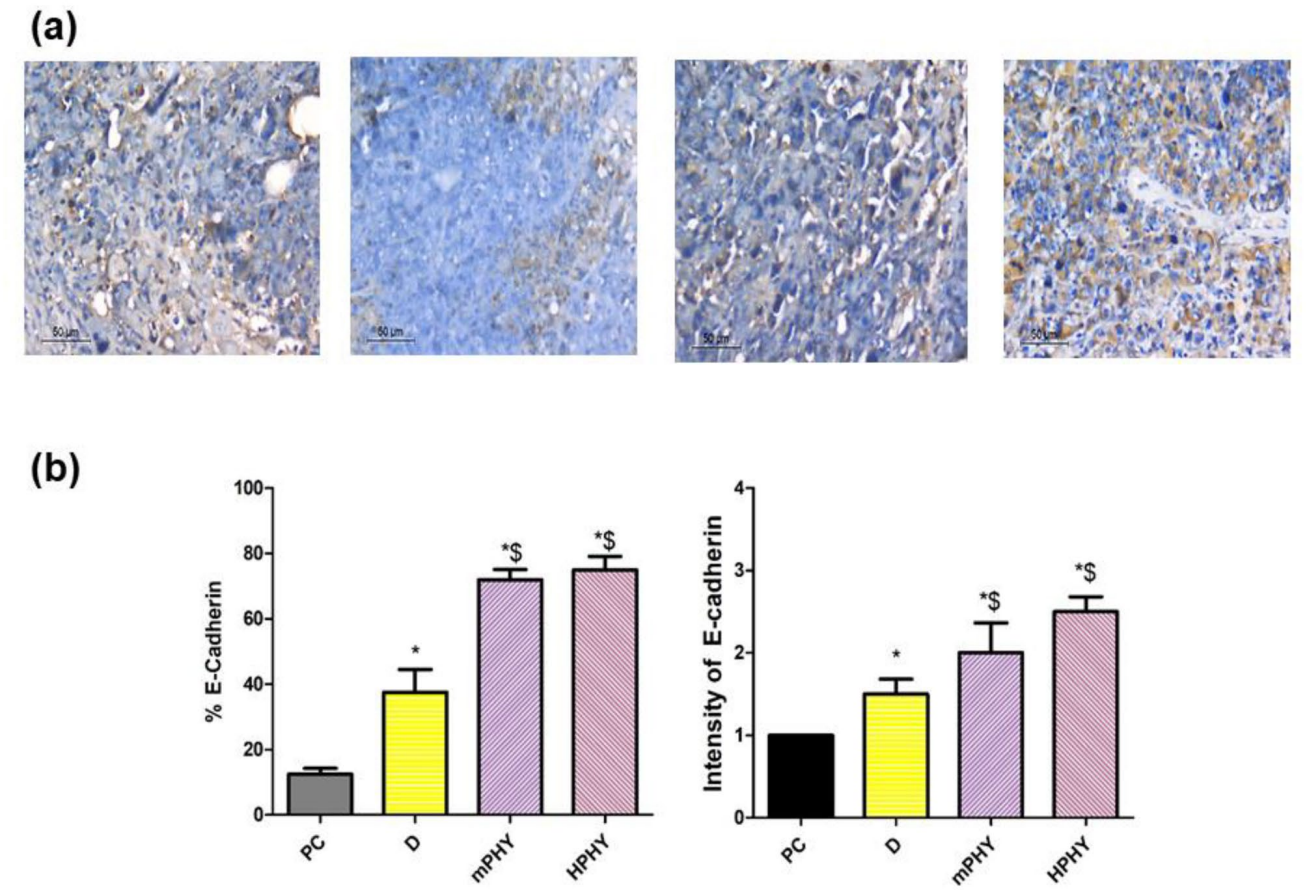
Effect of FIS (D) and modified cholephytosomes (mPHY and HPHY) on the immunohistochemical expression of E-cadherin in breast carcinoma: representative photomicrograph (X400) (**a**) and quantitative determination (**b**) of (I) percent immunostaining and (**II)** intensity of stain. PC untreated mice, mPHY cationic SA-bearing cholephytosomes, HPHY hyaluronic decorated cholephytosomes. Statistical analysis was done using one-way ANOVA followed by Student–Newman–Keuls multiple comparison test; *P < 0.05 vs PC, $*P* < 0.05 vs D

It is noteworthy that downregulation of TGF-β1/MMP signaling pathway has shown to be crucial in altering the progression of several types of cancer [79, 80]. Furthermore, inhibition of active MMP-9 could contribute in the early interruption of the metastatic circuit [81]. Inhibition of MMP-9 in breast cancer was reported to be achieved by interfering with the PKCα/ERK/AP-1/STAT3 [82] or the Akt/NF-κB [83] signaling pathways. In case of fisetin, reduction of MMP-9 expression in breast cancer cells was attributed to several molecular pathways including primarily the suppressed activation of ERK1/2, ROS, p38 MAPK signaling, and also the NF-κB related pathways [84]. Recently, our research group came to confirm that fisetin and its encapsulated bioinspired lipid nanoparticles could halt cell cycle progression through a NF-κB/caspase-3 signaling pathway in animal model of breast cancer [85]. In the current study, free fisetin (drug solution, D) has shown an effective decrease of the TGF-β1 amount in breast cancer tissue, which was associated with decreased ERK1/2 and NF-κB, but not the MMP-9 amounts (Fig. 10). The inhibitory effect of FIS on TGF-β1 has been reported for liver carcinoma [86] and different inflammatory diseases [86, 87]. *To the* *best of our knowledge, this study come to first describe the effect of FIS and/or its encapsulated nanomedicine on TGF-β1 using EAC-induced breast cancer.* Besides, an increased amount of necrotic tissue was also observed upon histological staining (Fig. 11).

Using modified cholephytosomes (mPHY and HPHY) could markedly improve FIS pharmacological activity against breast cancer cells. This was clearly manifested for both nanovesicles through increased necrotic tissue amount (Fig. 11), alongside with decreased level of ERK1/2, NF-κB, MMP-9, and hence, TGF-β1 (Figs. 9 and 10). The significant inhibition of tumor biomarkers by modified cholephytosomes could confirm the ability of both stearylamine bearing cholephytosomes (mPHY) and hyaluronic decorated cholephytosomes (HPHY) to enhance FIS bioavailability and to deliver it efficiently into tumor site emphasizing its anticancer effect through multiple pathways including MMP-9. Such result could be attributed to targeting potential of both stearylamine (SA) and hyaluronic acid (HA) to the externalized phosphatidylserine (PSe) and the overexpressed CD-44 receptors on breast cancer cells, respectively. Moreover, the PEGylated surfactant, TPGS, could also impart cholephytosomes stealth properties preventing their uptake by reticuloendothelial system and subsequently promote their accumulation into tumor site via their targeting ligands.

Compared to SA-bearing cholephytosomes (mPHY), HA-decorated cholephytosomes (HPHY) could significantly decrease the ERK1/2, NF-κB, and MMP-9 levels (*P* < 0.05) as seen in Fig. 10. While, mPHY exhibited superior inhibitory effect on TGF-β1 compared with HPHY (*P* < 0.05) as depicted in Fig. 9. Such result could be attributed to the selective interaction of stearylamine (SA) with surface exposed phosphatidylserine (PSe) which was reported to contribute in tumor invasion and metastasis through increasing TGF-β1 level. On other word, binding of SA with PSe could partially interfere with TGF-β1 secretion leading to a synergistic TGF-β1 inhibitory effect to FIS as was confirmed through immunohistochemical staining of breast cancer tissue [34, 88]. Such outcome could be mediated via other TGF-β1 signaling pathways such as the canonical SMAD-dependent pathway and other non-canonical pathways including PI3K/Akt, and ROS/MAPK signaling [72].

#### Effect of FIS-cholephytosomes on E-cadherin

E–cadherin is one of the epithelial markers that are down-regulated during epithelial-mesenchymal transition (EMT) that occurred in different epithelial cancers including breast cancer. In EMT process, the cancerous epithelial cells lose their adherent and tight junctions detaching from their neighbors and gain mesenchymal properties resulting subsequently into cancer progression, invasion, and metastasis [89]. Recently, FIS has been demonstrated to reverse the epithelial to mesenchymal transition and subsequently reduced breast cancer progression and metastasis in vivo using triple negative breast cancer xenograft model bearing MDA-MB-231 cells [90]. *Yet, the effect of FIS on EMT has not investigated so far on Ehrlich ascites induced breast cancer model.* Therefore, E–cadherin expression was assessed in the current study to investigate the effect of FIS on EMT process in comparison with modified FIS-cholephytosomes.

The results of the E-cadherin immunostaining revealed the potential of fisetin phytochemical to interrupt EMT through upregulation of the epithelial marker E-cadherin. Furthermore, these results came to confirm the superior anti-tumoral effect of FIS modified cholephytosomes, where both mPHY and HPHY have shown an abrupt increase in the amount of E-cadherin in comparison with free drug (*P* < 0.05), Fig. 11. It is well recognized that upregulation of epithelial marker E-cadherin is related to a decrease in tumor aggressiveness and progression affecting many aspects such as adhesion, transformation, angiogenesis, and metastasis [91]. Several studies believe that the main player of suppressed E-cadherin-related tumor metastasis is the increased MMP-9 levels [92, 93]. The well-elucidated mechanism by which increased MMP-9 could cause degradation of E-cadherin is the regulation of the Wnt/β-catenin [94] and FGFR/MEK/ERK pathways [92]. Thus, targeting E-cadherin-associated MMP-9 pathway with modified cholephytosomes can be a promising therapeutic target for breast cancer metastasis and invasion as elucidated in the current study.

#### Histopathological examinations

Histopathological examination of breast tissues revealed high grade invasive mammary carcinoma with minimal infiltrative necrosis in untreated mice. Treatment with fisetin either as free form (D) or as nanocomplexes (mPHY and HPHY) showed higher values of necrosis within carcinoma cells, highlighting its anti-tumoral potential as phytochemical (Fig. S3). In spite of the promising results of biochemical assay and immunohistochemical staining that manifested obviously the outperforming of modified FIS-cholephytosomes over free FIS, the histopathological examination could not reveal any difference between free FIS and its nanovesicles. This because necrosis is not the main cytotoxicity mechanism of FIS against the investigated breast cancer model as was asserted by our team in an earlier study [85].

The histological examination of the liver and kidney tissues could not detect pathological changes in the examined the liver and renal tissues of both free drug and HPHY treated groups. mPHY showed mild histological changes on tissues of liver including moderate portal inflammation, congestion, lobular activity, feathery degeneration of hepatocytes and giant cells, while renal tissue featured mild interstitial congestion (Fig. S4).

Overall, in vivo studies confirmed the ability of both cationic SA-bearing cholephytosomes (mPHY) and hyaluronic decorated cholephytosomes (HPHY) to slow down breast tumor progression by upregulating the epithelial marker E-cadherin in addition to downregulating TGF-β1 level and its non-canonical related signaling pathway; ERK1/2, NF-κB, and MMP-9. This was accompanied by increased necrosis area and better pathological profile. Eventually, such work come to spotlight the potential of cationic SA-dependent nanovesicles to perform as promising nanocarriers for targeting FIS into breast cancer in a comparable manner to the well-known CD-44 targeting approach. Meanwhile, their systemic toxicity should be considered before stepping up to clinical settings.

## Conclusion

In this work, “PHYTOSOME” approach has been presented for the first time with fisetin (FIS) to allow its intravenous administration to breast cancer. In addition, cholesterol was incorporated to FIS-phospholipid mixture to tailor novel cholephytosomes capable of improving FIS pharmaceutical properties as well as its anticancer activity against breast cancer. Then, modified cholephytosomes were prepared by two different approaches; charge-dependent and receptor mediated approaches to improve FIS delivery to breast cancer. Stearylamine (SA) was utilized to develop cationic nanovesicles to orient the nanovesicles to the tumor externalized phosphatidylserine, while hyaluronic acid (HA) was used to develop CD-44 targeting nanovesicles. Modification of cholephytosomes was carried out in the presence of the bioactive surfactant TPGS acting as stabilizer in addition to its reported antitumor activity. Both cationic cholephytosomes and CD-44 targeting nanovesicles were appraised for their pharmaceutical performance and also their antitumor activity. Both of prepared cholephytosomes showed promising physicochemical properties including proper particle size (> 300 nm), suitable zeta potential (> ± 20mV), excellent FIS complexation efficiency (˷100%), improved n-octanol, and water solubility along with a sustained drug release over 24 h. Furthermore, Infrared spectroscopy and transmission electron microscopy have confirmed the interaction between drug and phospholipid and their spherical shape, respectively. In vitro cell line studies against MDA-MB-231 cell line revealed about 10-fold inhibition in IC50 of targeted FIS-cholephytosomes compared with free drug. Moreover, apoptosis was proved to be the predominant cytotoxicity mechanism of tested FIS-cholephytosomes as demonstrated by the flow cytometry study using annexin-V/propidium iodide assay. Intriguingly, the intravenous (IV) dose utilized in the in vivo animal studies (10 mg/kg) was 4 times lower than IV dose applied in literature against breast cancer. Nevertheless, modified FIS-phytosomes (mPHY and HPHY) could successfully suppress the tumor growth. Modulating TGF-β1and its non-canonical related signaling pathways have played a critical role in their anti-tumoral potential against mammary carcinoma. Overall, tailoring modified cholesterol integrated phytosomes for FIS in this work could successfully improve FIS physicochemical properties and emphasize its ant-tumor activity against breast cancer. Also, SA-bearing cholephytosomes exhibited comparable therapeutic efficacy in comparison with CD-44 targeting cholephytosomes. Meanwhile, ongoing and future studies should be focused on conducting pharmacokinetics and biodistribution studies for better appraisal and interpretation of pharmacological properties of FIS-integrated phytosomes. Also, full toxicological studies should be applied using different doses and by other routes of administrations particularly on cationic phytosomes to assert their systemic safety. This could ultimately pave the way for the probability of utilizing FIS in combination with other chemotherapeutic drugs in attempt to potentiate therapeutic outcomes of cancer patients along with minimizing systemic toxicity.

### Supplementary Information

Below is the link to the electronic supplementary material.

Supplementary file1 (DOCX 1981 KB)

## Data Availability

Not applicable.
