# Supplementary material for: Novel PEGylated cholephytosomes for targeting fisetin to breast cancer: in vitro appraisal and in vivo antitumoral studies
Source: Drug Deliv Transl Res. 2023 Aug 30;14(2):433–54. doi: 10.1007/s13346-023-01409-5 (PMC10761494; doi:10.1007/s13346-023-01409-5)
Supplement: Supplementary file 1 — Supplementary file1 (DOCX 1981 KB) [file 13346_2023_1409_MOESM1_ESM.docx]

Novel PEGylated Cholephytosomes for targeting Fisetin to Breast cancer: In-vitro appraisal and In-vivo antitumoral studies.

**Sara M. Talaat^1^, Yosra S.R. Elnaggar ^1,2*^,** **Mennatallah A. Gowayed^3^,** **Samar O. El-Ganainy****^3^**, **Maram Allam^4^, Ossama Y. Abdallah^1^**

1 *Department of Pharmaceutics, Faculty of Pharmacy, Alexandria University, Egypt.*

2 *Head of International Publication and Nanotechnology Center INCC, Department of Pharmaceutics, Faculty of Pharmacy and Drug Manufacturing, Pharos University of Alexandria, Egypt.*

*3 Department of Pharmacology and Therapeutics, Faculty of Pharmacy, Pharos University in Alexandria, Alexandria, Egypt.*

*4 Department of Pathology, Faculty of Medicine, Alexandria University, Alexandria, Egypt.*

****Correspondence:*** [yosra.s.elnaggar@gmail.com](mailto:yosra.s.elnaggar@gmail.com)

***In vivo antitumor activity study (section 2.6 in Methodology)***

Sections at the breast mass (mammary carcinoma) on coated slides were obtained from the prepared paraffin blocks. Sections were first deparaffinized using xylene, and descending concentrations of ethanol (100%, 95% and 70%) for 3 min at each step. After deparaffinization, antigen retrieval was carried out. Then slides were washed with 1% BSA with gentle agitation. The sections were then blocked in 10% normal serum with 1% BSA in TBS and incubated for 2 h for room temperature. Sections were then incubated in primary antibody; anti-mouse TGB-1 (a marker of tumor proliferation), clone, monoclonal antibody (1:50) in TBS with 1% BSA overnight at 4℃. After washing with TBS containing 0.025% triton 100X with gentle agitation the sections were incubated with anti-mouse HRP IgG conjugated antibody (1:40,000) in TBS with 1% BSA for 2 h at room temperature. Immunoreactivity was visualized after incubation with DAB for 10 min at room temperature followed by hematoxylin staining for 10 min and dehydration in the alcohol series using an Olympus microscope at high magnification. Immunohistochemical staining for TGB-1 was assessed as regards; the percent of positively stained cells (which was assessed using a computerized image analysis program LEICA SUIT) and intensity of positivity which scored (1= weak positivity, 2= moderately intense positivity, 3= markedly intense positivity).

***Fourier transform infrared spectroscopy (FTIR) (Section 3.5 in Results & Discussion)***

As seen in **Figure S1,** the FIS spectrum (a) showed several intense bands in the wave number region between 4000 and 400 cm^-1^. Fisetin (FIS) is characterized by absorption bands appearing at 3521 and 3352 cm^−1^ due to O-H stretching attached to aromatic rings of compound, 1607 cm^−1^ due to C=O stretching which is participated in the intra-molecular hydrogen bonding, 1569 cm^−1^(C=C stretching), 1477 cm^−1^(C-O stretching) and 1273 cm^−1^(C-O-H bending) (57). While SPC spectrum (b) exhibits characteristic peaks at 1735.5 cm−1 (C=O stretching), 1238 cm−1 (P–O asymmetric stretching), 1058 cm−1 (C–O–P stretching), and 968 cm−1 (N^+^(CH3)3 stretching) (56). The spectrum of the physical mixture of FIS to SPC in 1:1 molar ratio (c) showed an additive effect of FIS and SPC with slight changes.

Compared with physical mixture spectrum, the spectra of conventional FIS-phytosomes (D) and the modified cholephytosomes (E & F) showed marked spectroscopic changes in the characteristic peaks of their components. For example, PO_4_ group is mainly involved in interaction between phytomedicines and phospholipids. There was an observed shifting in two characteristic wavelengths corresponding to PO_4_ group stretching (1238 cm^-1^ & 1058 cm^-1^) in all vesicles’ spectra. Another shift was also manifested with the characteristic peak at 968 cm^−1^ corresponding to N^+^(CH3)_3_ stretching. Such shifts could be occurred due to interaction of FIS with the polar groups of the phospholipid (phosphate and amino groups). In addition, the broadening of OH band at 3352 cm^-1^ in all FIS/SPC complex spectra could indicate the presence of H-bonding interaction between phenolic-OH group of FIS and polar groups of SPC resulting in their complexation.


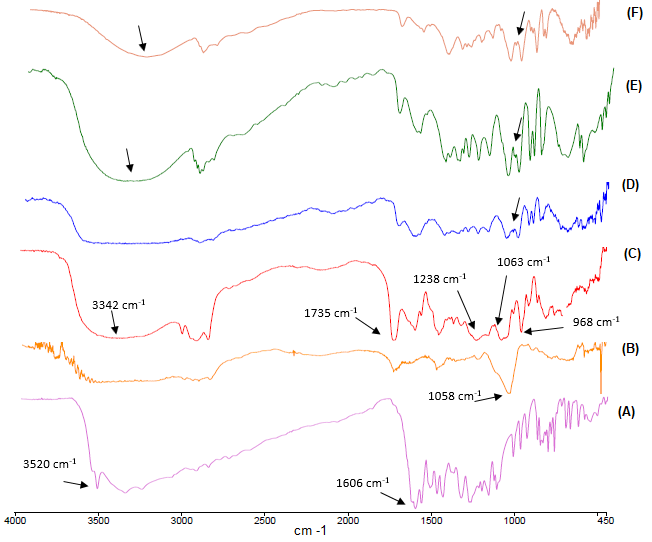


**Fig**. **S1** FTIR spectra of FIS (A), phospholipid SPC (B), physical mixture of FIS:SPC (1:1; C), PLX (D), HPHY (E), mPHY (F). Abbreviations: FTIR, Fourier transform infrared; FIS, Fisetin; SPC, soy phosphatidylcholine, PLX: conventional phytosomes, HPHY: hyaluronic coated cholephytosomes, mPHY: Cationic stearylamine bearing cholephytosomes

***Hemocompatibility test (Section 3.8 in Results & Discussion)***


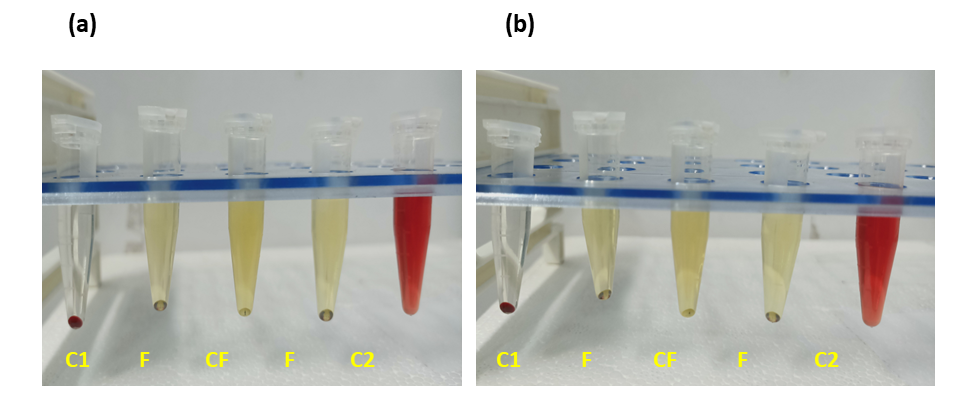


**Fig S2** Hemocompatibility test of FIS-loaded cholephytosomes. A) mPHY, B) HPHY. Note C1: saline (negative control), C2: 100% Triton X (positive control), F: tested nanoformulation, CF: control nanoformulation (RBCs-free formulation enrolled in the study as another control for each corresponding tested nanoformulation to allow visual inspection of any possible change in the color of the nanoformulation after addition of RBCs and thus, facilitating the comparison based on physical appearance)

***Stability of modified cholephytosomes in dried form (Section 3.4.6 in Results & Discussion)***

**Table S1. Physicochemical properties of freeze-dried modified fisetin cholephytosomes**

| Parameter | Freeze dried cholephytosomes* | |
| --- | --- | --- |
|  | **mPHY** | **HPHY** |
| Particle size (nm) | 316.40 ± 5.62 | 392.34 ± 8.17 |
| PDI | 0.45 ± .06 | 0.64 ± 0.08 |
| Zeta Potential (Mv) | + 40.54 ± 2.01 | -19.17 ± 1.20 |
| Entrapment Efficiency % | 97.84± 1.78 | 98.50 ± 0.41 |
| *Lyophilized in presence of 2% (w/v) mannitol and reconstituted directly after lyophilization. Abbreviations: mPHY: modified cationic fisetin cholephytosomes, HPHY: hyaluronic decorated fisetin cholephytosomes, PDI: poly dispersity index, Results of particle size measured as mean ± SD (n=3). | | |

***Histopathological Examinations (Section 3.11 in Results & Discussion)***


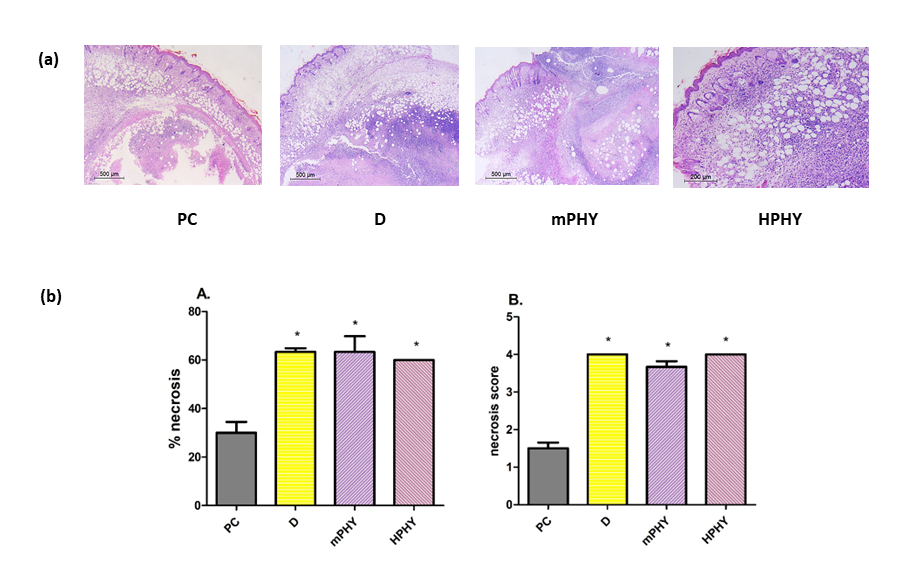


**Fig S3** FIS (D) and modified cholephytosomes (mPHY and HPHY) on Histopathological examination of breast tissues (X400): **I.** Representative photomicrograph of H&E stain (H&E Stain, x50**), II.** Quantitative determination of **A.** percent necrosis**, B.** Necrosis stain**.** PC: untreated mice, mPHY: cationic SA-bearing cholephytosomes, HPHY: hyaluronic decorated cholephytosomes. Statistical analysis was done using one-way ANOVA followed by Student-Newman-Keuls multiple comparison test; *p<0.05 vs PC


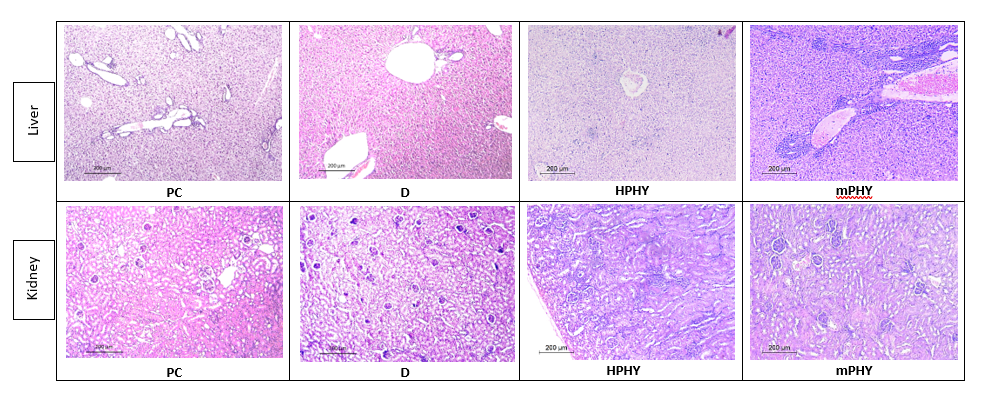


**Fig S4** Representative photomicrographs of H&E stain (H&E Stain, x50) showing the effect of modified cholephytosomes (mPHY and HPHY) on liver and kidney tissues (X400) in comparison with FIS (D) and positive control (PC)
